# Exploring covalent inhibitors of SARS-CoV-2 main protease: from peptidomimetics to novel scaffolds

**DOI:** 10.1080/14756366.2025.2460045

**Published:** 2025-02-06

**Authors:** Noor Atatreh, Radwa E. Mahgoub, Mohammad A. Ghattas

**Affiliations:** ^a^ College of Pharmacy, Al Ain University, Abu Dhabi, United Arab Emirates; ^b^ AAU Health and Biomedical Research Centre, Al Ain University, Abu Dhabi, United Arab Emirates

**Keywords:** Anti-viral, irreversible inhibition, covalent inhibitor, Covid-19, warheads reactivity

## Abstract

Peptidomimetic inhibitors mimic natural peptide substrates, employing electrophilic warheads to covalently interact with the catalytic Cys145 of M^pro^. Examples include aldehydes, α-ketoamides, and aza-peptides, with discussions on their mechanisms of action, potency, and structural insights. Non-peptidomimetic inhibitors utilise diverse scaffolds and mechanisms, achieving covalent modification of M^pro^.

## Graphical abstract

**Figure:**
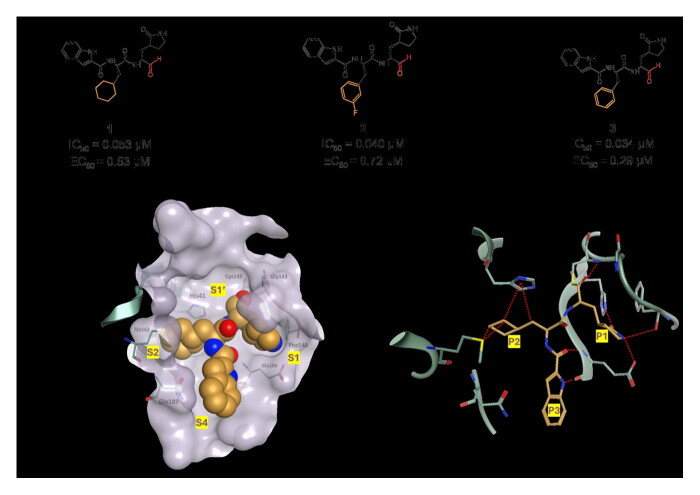

## Introduction

In December 2019, a novel coronavirus (SARS-CoV-2) emerged, leading to the outbreak of Coronavirus Disease 2019 (COVID-19), which the World Health Organisation (WHO) declared a global pandemic on March 11, 2020^1^^,^^2^. Despite having a lower case fatality rate (CFR) of 2–3% compared to SARS-CoV in 2002 and MERS-CoV in 2012, SARS-CoV-2’s high transmissibility and severe impact on immunocompromised individuals resulted in significant challenges in controlling its spread, leading to a high number of fatalities^3^^,^^4^. As of January 5, 2025, there have been 777 126 421 confirmed cases and 7 079 925 deaths globally according to WHO^5^. These repeated outbreaks of coronaviruses highlight the urgent need for enhanced global health preparedness and a better understanding of zoonotic transmission dynamics, making the scientific community prioritise developing strategies to prevent future zoonotic outbreaks.

The SARS-CoV-2 main protease (M^pro^), also known as 3-chymotrypsin-like cysteine protease (3CL^pro^), is a critical target for drug development due to its role in cleaving polyproteins to produce 12 non-structural proteins necessary for viral replication^6^^,^^7^. M^pro^ specifically recognises the cleavage sequence Leu – Gln ↓ – X (X = Ser/Ala/Gly), with the cleavage site indicated by ↓^8^. This specificity, distinct from human proteases, makes M^pro^ an attractive target for inhibition, reducing potential off-target effects. Research is focused on developing inhibitors that bind to M^pro^’s active site, blocking its function and inhibiting viral replication^9^.

## SARS-CoV-2 M^pro^ structure

**Figure 1.**
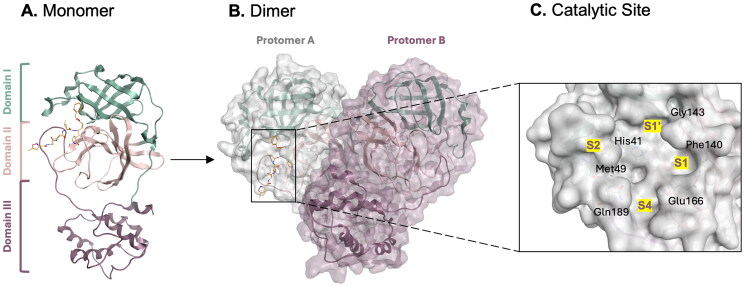
SARS-CoV-2 M^pro^ **A.** monomer form (PDB: 6LU7)^8^ and **B.** dimer form (PDB: 7CAM). **C.**^11^ A close-up view of the catalytic binding site made up of 4 subsites. This figure was produced by the authors.

The SARS-CoV-2 M^pro^ enzyme is a 33.8 kDa cysteine protease dimer, made up of two protomers each featuring three distinct domains: I (residues 8–101), II (residues 102–184), and III (residues 201–306) (Figure 1A and ^B^)^6^^,^^10^. Domains I and II exhibit a chymotrypsin-like structure with a six-stranded antiparallel β-barrel fold, while domain III comprises five antiparallel α-helices^6^^,^^10^^,^^12^, playing a crucial role in the enzyme’s catalytic activity through dimerisation via an intermolecular salt-bridge interaction between Glu290 and Arg4^13^^,^^14^. The dimer state, with an estimated dissociation constant of ∼2.5 µM, is optimal for hydrolytic activity^6^^,^^10^.

The SARS-CoV-2 M^pro^ structure is 96% similar to the SARS-CoV M^pro^ structure, differing in only 12 amino acid residues^9^^,^^15^. The SARS-CoV M^pro^ dimer has a polar interaction between domains III mediated by a H-bond and hydrophobic contacts, maintaining dimer unity. In contrast, SARS-CoV-2 M^pro^ has Ala and Leu residues in place of Thr and Ile involved in dimer maintenance interactions, resulting in a closer distance between the two domains III^8^^,^^10^, leading to enhanced catalytic activity^16^ and potentially contributing to the increased contagiousness of SARS-CoV-2.

The substrate-binding site is made up of four sub-pockets (S1, S2, S4, and S1′) (Figure 1C) ^17^, located in a deep cleft between domains I and II^10^, with the N-terminal finger (domain I, residues 1–7) being important for catalysis^12^^,^^18^. The S1′ sub-site contains residues His41 and Cys145, making up the catalytic dyad^19^ as well as a third catalytic element in the form of a conserved buried water molecule^19–21^, aiding in catalysis by forming a H-bond with His41^22^. Each protomer has one substrate binding site, with the N-terminal of each protomer interacting with Glu166 of the other protomer to shape and stabilise the S1 sub-pocket^22^, requiring dimerisation for optimal catalytic activity. Given the importance of the S1 sub-pocket in binding the conserved glutamine residue of the polyprotein substrate, it is crucial to target the residues in this sub-pocket when developing antiviral drugs against M^pro^^23^.

**Figure 2.**
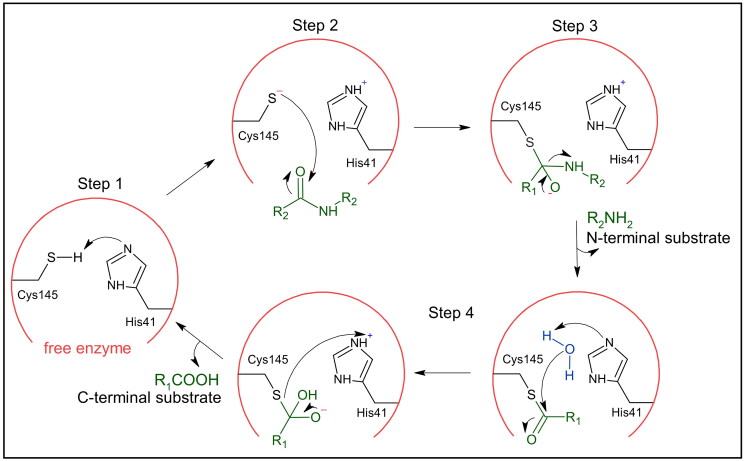
Hydrolysis mechanism of SARS-CoV-2 M^pro^. Residues of the catalytic dyad and substrate are depicted in black and green, respectively. This figure was produced by the authors.

The M^pro^ enzymatic activity depends on the catalytic dyad comprising Cys145 and His41, with Cys145 serving as the hydrolysis agent via its thiol (-SH) group, while His41 maintains optimal pH conditions for the activation of the thiol group^19^^,^^21^. The process involves four steps (Figure 2): (1) deprotonation of the -SH group by His41 leading to the formation of an activated thiolate ion, (2) nucleophilic attack by the activated thiolate ion on the carbonyl carbon of the substrate, resulting in the formation of a tetrahedral adduct. (3) release of the hydrolysed peptide product with a free N-terminus and regeneration of catalytic His41, (4) and hydrolysis of the thioester bond, resulting in the release of the remaining peptide fragment with a free C- terminus^24^^,^^25^.

In the scientific community, there’s ongoing debate about the mechanism of the enzymatic activity of the M^pro^ enzyme in SARS-CoV-2, focusing particularly on whether the deprotonation of the thiol group in Cys145 by His41 and the subsequent attack by the thiolate ion occur sequentially or concertedly. The sequential theory posits that the proton transfer from Cys145 to His41 is an initial low-energy barrier event, facilitating a quick shift between the ion-pair and neutral states of the dyad^26^. This perspective is reinforced by other studies that computationally identify transition states where proton transfer completes prior to the nucleophilic attack, suggesting that the proton transfer is the preliminary, less demanding step^24^.

Conversely, another viewpoint advocates for a simultaneous, concerted mechanism where both the deprotonation and nucleophilic attack occur together. Here, the proton transfer is seen as energetically more challenging than the nucleophilic attack, serving as the rate-limiting step^27^. Once the enzyme overcomes this proton transfer free energy barrier, the nucleophilic attack promptly follows^27^. These mechanistic insights are essential for the strategic design of inhibitors targeting the M^pro^ enzyme. Understanding whether the enzyme’s actions are sequential or concerted can help pinpoint critical interaction points and guide the development of therapeutic compounds that effectively disrupt the virus’s lifecycle.

## SARS-CoV-2 M^pro^ as a target for drug design

M^pro^ has gained attention as a promising drug target against SARS-CoV-2 due to its unique cleavage pattern, which lowers the likelihood of toxicity compared to other targets. It has been reported that inhibition and alteration of the catalytic dyad made up of His41 and Cys145, would result in decreasing the catalytic activity of the enzyme, and affect the folding of the structure^28^. Compounds that form covalent bonds with the M^pro^ enzyme have been developed, leveraging their superior selectivity.

**Figure 3.**
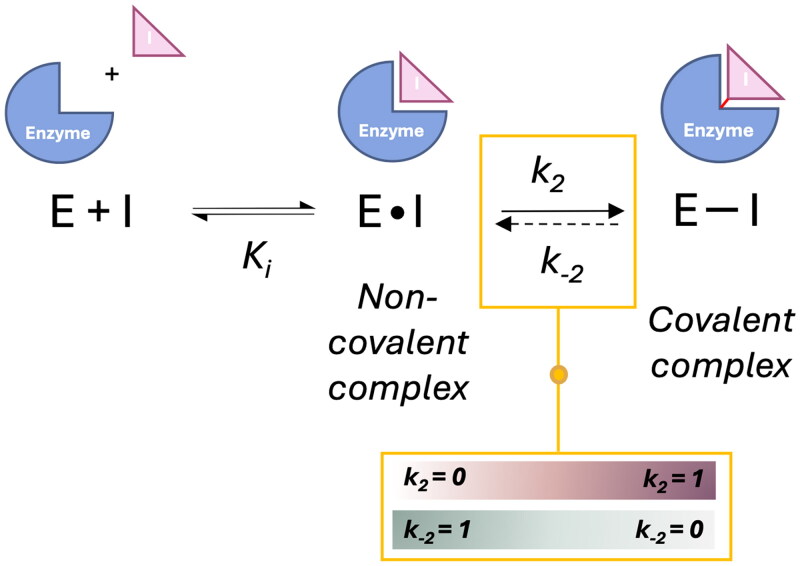
Stages and kinetic continuum of covalent complex formation. This Figure was produced by the authors.

The inhibition mechanism through covalent complex formation unfolds in two distinct phases (Figure 3): Initially, the inhibitor engages non-covalently with the amino acids in the binding pocket, positioning the warhead adjacent to the enzyme’s nucleophile, represented by the equilibrium constant K_i_. Subsequently, a covalent bond forms between the warhead and the target’s nucleophile, a step quantified by k_2_. The stability and reversibility of the resulting covalent complex are dictated by k_−2_, the rate at which the complex dissociates back to the non-covalent complex form. For inhibitors forming irreversible covalent complexes, this dissociation rate is zero (k_−2_ = 0), indicating a permanent covalent bond, while reversible inhibitors demonstrate measurable k_−2_, allowing transition back to non-covalent complex form. Non-covalent inhibitors lack covalent bonding capability, inherently assigning k_2_ = 0 to their interactions^29^^,^^30^.

**Figure 4.**
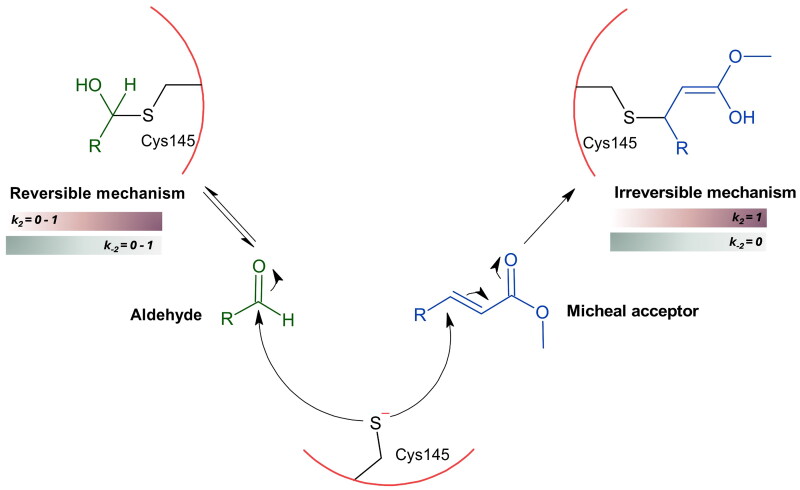
SARS-CoV-2 M^pro^ enzyme inhibition via reversible and irreversible covalent bond formation, depending on the warhead. This figure was produced by the authors.

In the case of the M^pro^ enzyme inhibition, two types of covalent inhibitors have been designed: reversible covalent inhibitors, as well as irreversible covalent inhibitors. The inhibition mechanism involves two stages: a reversible first stage where the inhibitor interacts with the binding pocket’s amino acids, followed by a second stage where the thiol group of Cys145 attacks the inhibitor’s most activated carbon atom, forming a new bond. The strength of the newly formed covalent bond determines the reversibility of inhibition, which can be restored by water or rendered permanent through irreversible binding (Figure 4). This is greatly influenced by the intrinsic reactivity of the functional group of the inhibitor. Michael acceptors are an example of warheads leading to irreversible inhibition, while aldehydes are an example of reversible covalent inhibitors. Peptidomimetics and non-peptidomimetics have shown significant biological activity *in-vitro* and are potential anti-COVID-19 drugs. A description of these compounds in terms of chemical scaffold, inhibition mechanism and biological activity is provided in the next section.

## Classifications of M^pro^ inhibitors

Covalent inhibitors of the SARS-CoV-2 M^pro^ have garnered significant attention from the scientific community, with the goal of designing drug-like inhibitors that form stable covalent bonds with the enzyme, thereby blocking its activity and inhibiting viral replication. These inhibitors can be classified based on various criteria, such as the nature of their reactive groups, mechanisms of action, or chemical scaffolds. This section will categorise reported M^pro^ covalent inhibitors into two major groups based on their chemical scaffolds: peptidomimetics and non-peptidomimetics.

### Peptidomimetic inhibitors of SARS-CoV-2 M^pro^

Peptidomimetics have been extensively researched as they mimic the native peptide substrate of the M^pro^ enzyme. This approach offers superior metabolic stability, pharmacokinetic, and potency profiles compared to natural peptides^31^, emerging as the most widely used approach for the development of anti-COVID-19 drugs^32^^,^^33^. The inhibitors’ structure includes P1’, P1, P2, and P3 components, with modifications to the peptide backbone (P1–P3) to enhance druglike properties and provide specific recognition motifs for SARS-CoV-2 M^pro^. The C-terminal electrophilic function (warhead) at the P1’ site is critical for covalent inactivation of the protease catalytic subsite (i.e. S1′). Some examples are summarised in Table 1. These warheads can be further classified into reversible and irreversible covalent bond inhibitors, based on the strength of the bond formed.

**Table 1.** Electrophilic warheads found in current peptidomimetic M^pro^ inhibitors.

	Electrophilic warhead	Covalent bond mechanism	Covalent bond reversibility	Warhead structure
	Aldehyde	Nucleophilic Addition to a Double Bond	Reversible	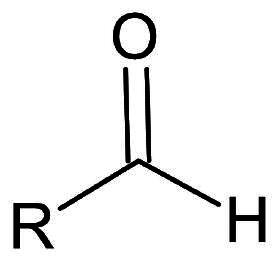
*Ketones*	Hydroxymethyl ketone	Nucleophilic Addition to a Double Bond	Reverisble	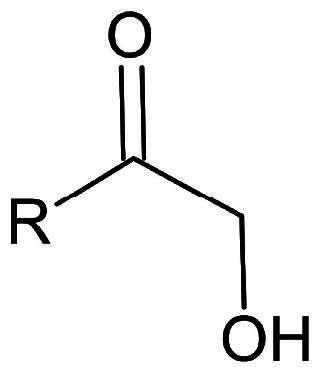
	Acyloxy methyl ketone	Nucleophilic Addition to a Double Bond	Irreversible	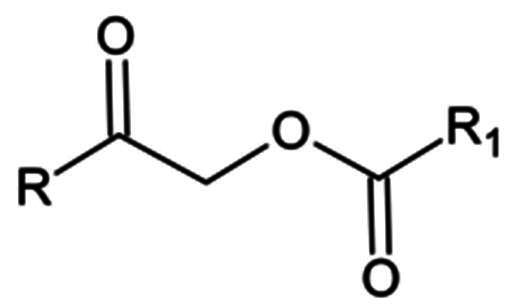
	Benzothiazole ketone	Nucleophilic Addition to a Double Bond	Reversible	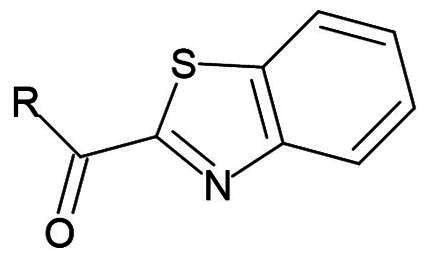
	Fluro benzothiazole ketone	Nucleophilic Addition to a Double Bond	Reversible	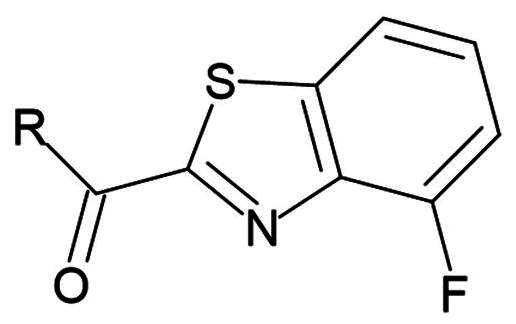
*Micheal acceptors*	Vinyl esters	Michael Addition	Irreversible	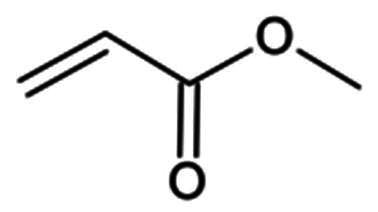
	Nitroalkene	Michael Addition	Reversible	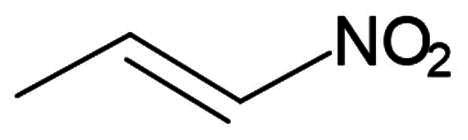
	α-Ketoamide	Nucleophilic Addition to a Double Bond	Reversible	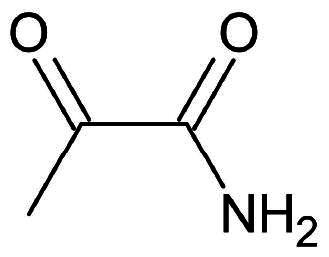
	2-Haloactamide	Nucleophilic Substitution	Irreversible	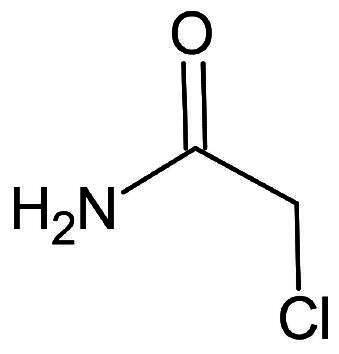
*Others*	Nitrile	Nucleophilic Addition to a Triple Bond	Reversible	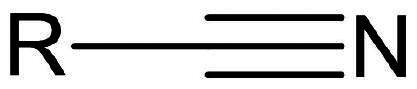
	Alkyne	Hydrothiolation	Irreversible	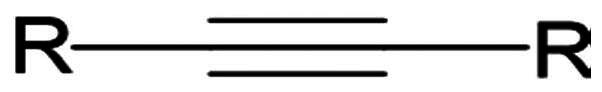
	Pyrazolidinone	**γ**-lactam ring opening	Not Investigated	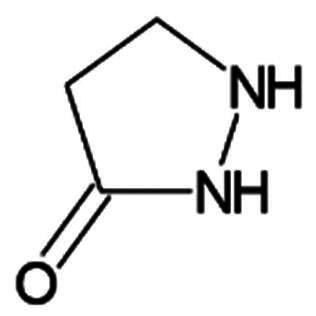

#### Aldehydes

Aldehydes are known for their reactivity and ability to form reversible covalent hemi-thioacetal adducts with cysteine residues^34–36^, particularly Cys145 in the active site of M^pro^. Peptidyl aldehydes in particular show promise as inhibitors due to their selectivity towards cysteine proteases. Their superior activity can be attributed to several factors, including their ability to maintain the carbonyl group at the P1 position of the peptide substrate. This positioning allows for crucial H-bond formation within the active site of the protease, enhancing both binding affinity and specificity. Therefore, peptidyl aldehydes represent a promising class of compounds for the development of inhibitors targeting SARS-CoV-2 M^pro^.

**Figure 5.**
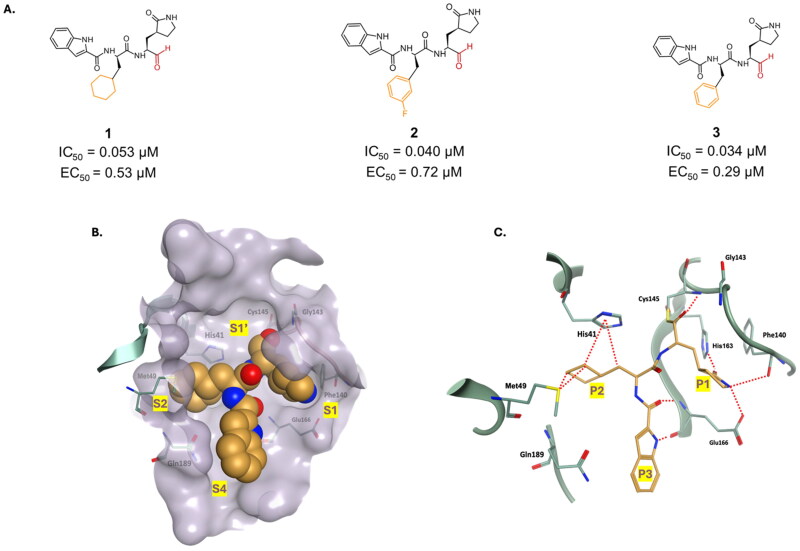
**A.** Chemical structures of peptidomimetic covalent inhibitors of SARS-CoV-2 M^pro^ with an aldehyde warhead depicted in red, and first-point modification depicted in orange. **B.** X-ray crystal structure of 6LZE, depicting the fitting of ligand **1** in the M^pro^’s active site. **C.** Ligand interaction of 6LZE complex. This figure was produced by the authors.

Compounds **1** and **2**, are synthetic aldehyde-bearing peptidomimetics (Figure 5A), with promising inhibitory activity against SARS-CoV-2 M^pro^, with a half-maximal inhibitory concentration (IC_50_) values of 0.053 µM and 0.040 µM, respectively^23^. Furthermore, they also exhibited strong antiviral activity in Vero E6 cells infected with SARS-CoV-2, leading to significantly reduced viral replication with half maximal effective concentration (EC_50_) values of 0.53 µM and 0.72 µM, respectively). Neither compound reported significant cytotoxicity, with half cytotoxic concentration (CC_50_) values of >100 μM. These compounds were designed based on successful inhibitors of the SARS-CoV M^pro^ and have been found to effectively target the M^pro^ of SARS-CoV-2 as well. The X-ray structures of compounds **1** and **2** (PDB: 6LZE and 6M0K, respectively)^23^ provide valuable insights into their fitting and interactions within the active site of the enzyme, aiding in the rational design of future inhibitors. The γ-lactam at P1 is fitted in the S1 sub-site, while the aliphatic side chain at P2 is housed within the hydrophobic S2 pocket (Figure 5B). The indole moiety at P3 is located at the surface interface, S4. As anticipated, both inhibitors were observed to form a covalent bond with the thiol group of Cys145 through their aldehydic carbonyl (Figure 5C). The additional H-bond formed by the aldehydic warhead with Cys145 backbone stabilised the covalent bond formed. The γ-lactam forms two H-bonds with Glu166, and His163, as well as an additional H-bond with Phe140. Finally, the aliphatic side chain in S2 is supported by several hydrophobic interactions. Using the scaffold of these potent inhibitors, a more potent analogue was optimised to house a benzyl group at the P2 position (Figure 5A), which led to compound **3**, with an improved IC_50_ (0.034 µM) and EC_50_ (0.29 µM) in Vero E6 cells. It also reported a CC_50_ > 100 µM (808 µM)^37^.

**Figure 6.**
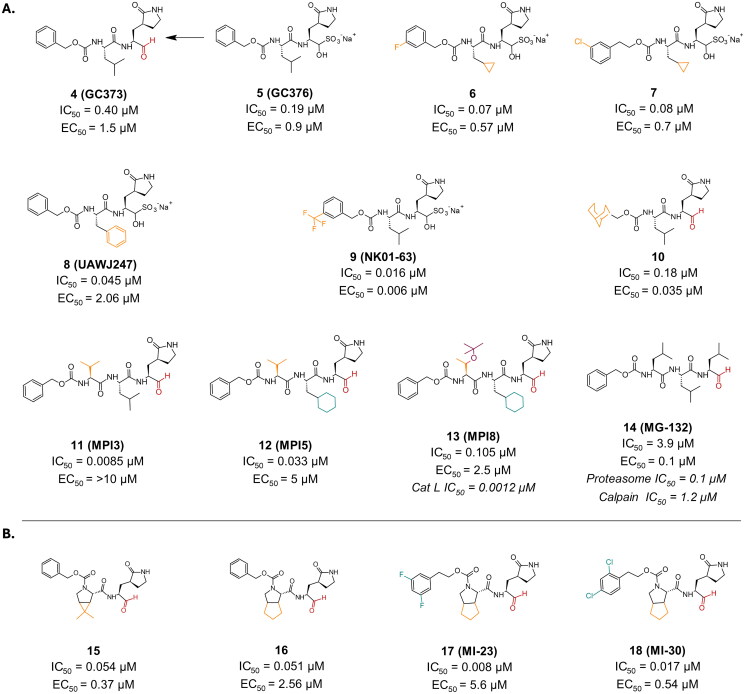
Chemical structures of **A.** GC373, and **B.** boceprevir and telaprevir peptidomimetic derivatives as covalent inhibitors of SARS-CoV-2 M^pro^ with an aldehyde warhead depicted in red, first-point modification depicted in orange, second-point modification depicted in blue, and third-point modification depicted in purple. This figure was produced by the authors.

Compound **4** (**GC373**), and its potent bisulphite prodrug compound **5** (**GC376**) are reversible peptidyl aldehyde inhibitors used against feline coronavirus (FCoV) (Figure 6A), known to induce fatal feline infectious peritonitis (FIP) in cats^38^. Interestingly, they were also found to be active against SARS-CoV-2 M^pro^ with IC_50_ values of 0.40 µM and 0.19 µM, respectively, and EC_50_ values of 1.5 µM and 0.9 µM, respectively, in Vero E6 cells. Notably, both compounds exhibit low cytotoxicity, with a CC_50_ > 100 µM^39^. The formation of the covalent bond was confirmed by crystallography (PDB: 6WTK and 6WTJ, respectively)^39^^,^^40^. The reversibility of the covalent bond formed by these compounds was investigated via a ^13^CHO-labelled compound **5** (GC376) and a time-dependent NMR experiment^41^. Compounds **6** and **7** are synthetic derivatives of compound **4** and **5** (Figure 6A). Compound **6** (PDB: 7LCO)^42^ and **7** features modifications at the P2 and P3 positions with cyclopropyl and meta-halophenyl substituents, respectively, leading to enhanced interactions and an improved IC_50_ value of 0.07 µM and 0.08 µM, respectively^42^. Their antiviral activity also exhibited a slight improvement with EC_50_ values of 0.57 µM and 0.7 µM, respectively, in Vero E6 cells. The EC_50_ is improved upon administration with the efflux inhibitor CP-100356 (0.19 µM and 0.18 µM for compounds 6 and 7, respectively). No cytotoxicity was observed up to >200 µM in Vero E6 cells^42^. More attempts of optimisation of compounds **4** and **5** resulted in derivatives **8** (**UAWJ247**), and **9** (**NK01-63**) (Figure 6A), with improved IC_50_ values of 0.045 µM and 0.016 µM, respectively^43^^,^^44^. The antiviral performance of compound **9** showed significant improvement, with an EC_50_ of 0.006 µM in Huh-7^ACE2^-infected cells, while compound 8 reported an EC_50_ of 2.06 µM in plaque assay conducted in Vero 6 cells. Compound 8 exhibited a CC_50_ of >100 µM in multiple cell lines (Caco-2, HCT, Huh-7, MDCK, and Vero cell lines), while compound 9 reported no *in-vivo* toxicity in *in-vivo* studies conducted in mice. Compound **10** (Figure 6A) is another example of derivatization attempts of compounds **4** and **5**, housing a constrained bicyclic ring system at P3, resulting in a slightly improved IC_50_ (0.18 µM). The *in-vivo* testing of **10** in Vero E6 cells reported an improved antiviral activity with EC_50_ of 0.035 µM^45^. Additionally, it reported a CC_50_ > 100 µM.

Through another attempt at optimisation of compound **4**, compounds **11** (**MPI3**), **12** (**MPI5**), and **13** (**MPI8**) depicted in Figure 6(A), were developed as aldehydic reversible tripeptide inhibitors of SARS-CoV-2 M^pro^ enzyme. Compound **11** had a significantly improved IC_50_ in the nanomolar range (0.0085 µM), while compounds **12** and **13** exhibited a significantly improved antiviral activity, with EC_50_ values of 5 µM and 2.5 µM, respectively^46^. Compound **13** was classified as a dual inhibitor as it also inhibited cathepsin L (IC_50_ = 0.0012 µM)^47^, which is suspected to facilitate coronaviral entry in the cell. This paved the way for the discovery of compound **14** (**MG-132**) via a repurposing study exploring known proteasome inhibitors (Proteasome IC_50_ = 0.1 µM and Calpain IC_50_ = 1.2 µM)^48^^,^^49^ (Figure 6A). Its reported IC_50_ against SARS-CoV-2 M^pro^ enzymes was 3.9 µM^50^, and an EC_50_ in Vero E6 cells of 0.1 µM^51^. However, compound 14 was found to be cytotoxic in all cell lines investigated with a CC_50_ ranging from 0.14 − 10.71 µM^50^.

Compounds **15,** and **16,** bearing an aldehyde functionality, were synthetic derivatives inspired by the FDA-approved antiviral agents **boceprevir** and **telaprevir**, as well as compound **4** (Figure 6B)^52^. These derivatives are an example of successful hybridisation approach, leading to a nanomolar inhibitory activity (IC_50_ of 0.054 µM and 0.051 µM, respectively) and a submicromolar antiviral activity (EC_50_ of 0.37 − 2.6 µM in Vero E6 cells).

Compound **17** (**MI-23**) was a result of an extensive SAR study, which focused on modifying P3 (Figure 6B). It exhibited far superior inhibition with IC_50_ of 0.008 µM. However, its antiviral activity was reduced, with an EC_50_ of 5.6 µM in Vero E6 cells. In an effort to improve the antiviral activity, compound **18** (**MI-30**) (Figure 6B), featuring a 2,4-dichlorophenoxy acetal moiety at the P3 position, was investigated. Its exhibited IC_50_ value of 0.017 µM, which was worse compared to compound **17**. However, a notable improvement in the antiviral activity was achieved, with an EC_50_ value of 0.54 µM in Vero E6 cells^53^. They also showed a CC_50_ of >500 µM in HPAEpiC, LO2, BEAS-2B, A549 Huh7, and Vero 6 cell lines.

**Figure 7.**
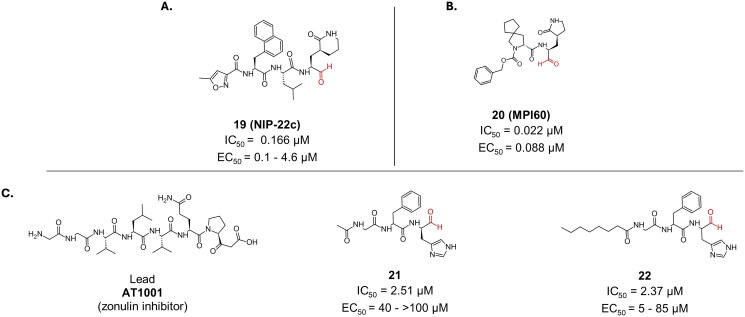
Chemical structures of **A.** di-peptidomimetic and **B.** tri- peptidomimetic covalent inhibitors of SARS-CoV-2 M^pro^ with an aldehyde warhead depicted in red. This figure was produced by the authors.

Compound **19** (**NIP-22c)** is a novel peptidomimetic potent inhibitor (Figure 7A) whose design was inspired by known peptidomimetics that have demonstrated significant efficacy against SARS-CoV-2^54^. The compound’s structural features include a naphthyl Group at the P3 position, an isobutyl Group at the P2 position, and a piperidone Ring at the P1 position, coining the name NIP. Compound **19** (**NIP-22c**) exhibited an IC_50_ of 0.166 µM, and varying EC_50_ values, ranging from 0.1 to 4.6 µM, depending on the cell model used. Importantly, compound **19** (**NIP-22c**) has been shown to retain its efficacy against multiple SARS-CoV-2 variants. Specifically, it has an EC_50_ value of 0.13 µM against the original Washington strain and an even lower EC_50_ value of 0.08 µM against the Omicron strain. These results highlight compound **19**’s potential as a robust antiviral agent capable of targeting diverse SARS-CoV-2 variants.

Compound **20** (**MPI60**) is a dipeptidyl M^pro^ covalent inhibitor, with an aldehydic warhead (Figure 7A), and a reported IC_50_ value of 0.022 µM, as well as a promising cellular EC_50_ of 0.088 µM^55^. Additionally, it portrayed low toxicity, with a CC_50_ of 95 µM. Crystallography has confirmed the covalent bond formation (PDB: 8STY)^55^.

Compound **21** is a synthetic tripeptide inhibitor with an aldehydic warhead, designed as a derivative of the zonulin inhibitor AT1001 (Figure 7B)^56^. Among the series of synthesised derivatives, compound **21** demonstrated the best inhibitory activity against the SARS-CoV-2 M^pro^, with an IC_50_ value of 2.51 µM. The covalent bond formation was confirmed by crystallography (PDB: 7ZV5)^56^. Compound **21** was also tested in Vero cells against three different strains of the SARS-CoV-2 (UK, Wuhan, and South African). It reported modest antiviral activity against the UK and Wuhan strains, with EC_50_ values of 40.3 µM and 66.1 µM, respectively. Its activity against the South African strain was rather weak with an EC_50_ > 100 µM. This level of activity was attributed towards the possibly compromised cell membrane permeability. Accordingly, compound **22** was synthesised by modifying the P4 position to introduce an octanoyl (Figure 7C). While this modification had an almost negligible effect on the IC_50_ (2.37 µM), it had a dramatic effect on the antiviral profile against the investigated strains, reporting EC_50_ values of 5 µM against both the UK and Wuhan strains, and a slightly improved EC_50_ in the sake of the South African strain (84.9 µM). Both synthesised peptides reported a CC_50_ of >100 µM against all three strains.

#### Ketones

Ketone-based inhibitors react with the nucleophilic thiolate group of Cys145 in the active site of the proteases, to form a hemi thioketal adduct. Hydroxymethyl ketones, and acyloxy methyl ketones, among other ketone-based warheads have been investigated as potential inhibitors of the SARS-CoV-2 M^pro^ enzyme. While hydroxymethyl ketones mainly act via reversible inhibition, α-acyloxy methyl ketones contrarily appear to be irreversible inhibitors. There are also some potent inhibitors with benzothiazole ketones, and Fluro—benzothiazole ketones warheads, forming irreversible covalent bonds.

##### Hydroxymethyl ketones

**Figure 8.**
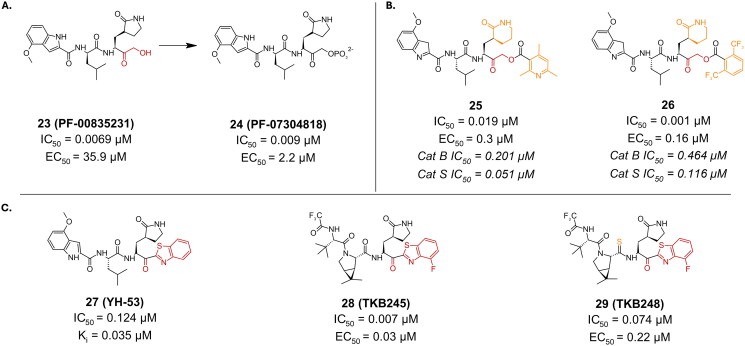
Chemical structures of peptidomimetic covalent inhibitors of SARS-CoV-2 M^pro^ with **A.** a hydroxymethyl ketone, **B.** an α-acyloxymethyl ketone, and **C.** a benzothiazole ketone warhead depicted in red, and first-point modification depicted in orange. This figure was produced by the authors.

Hydroxymethyl ketones emerged as potent inhibitors of the SARS-CoV-2 M^pro^. Compound **23** (**PF-00835231**), bearing a hydroxymethyl ketone warhead (Figure 8A), was among the first ketone-based inhibitors investigated, exhibiting an IC_50_ of 0.0069 µM, and an antiviral activity (EC_50_) of 35.9 µM in Vero E6 cells^57^. Its EC_50_ value is greatly improved (∼ 80 folds) once an efflux inhibitor is added, making it as low as 0.46 µM. To overcome some of its solubility issues and low oral bioavailability, **23** was transformed into compound **24** (**PF-07304814**) (Figure 8A), a phosphate prodrug, making it suitable for intravenous injection^58^.

##### α-acycloxymethyl ketones

Compounds **25** and **26** are potent examples where the C-terminal alcohol of **23** was extended using an alkyl or aryl group (Figure 8B), resulting in an α-acycloxymethyl ketone warhead, that forms an irreversible covalent bond upon reaction with the nucleophilic residue^59^. The IC_50_ values of these compounds were 0.019 µM and 0.001 µM, respectively. The covalent bond formation was confirmed via crystallography (compound **25** PDB: 7MBI)^59^. The antiviral activity investigated in Vero E6 cells for both compounds is also rather impressive, reporting EC_50_ values of 0.3 µM and 0.16 µM, respectively. Additionally, there seems to be no reported cytotoxicity up to 200 µM. It is also worth noting that while these compounds are highly selective towards cysteine proteases, they do exhibit activity towards human cathepsin B and S, with IC_50_ values of 0.201 µM and 0.051 µM, respectively, in the case of compound **25**, and 0.464 µM and 0.116 µM, respectively, in the case of compound **26**.

##### Benzothiazole

Compound **27** (**YH-53**) is a synthetic peptidomimetic leveraging a unique benzothiazole warhead (Figure 8C)^60^. This compound was developed based on a tetrapeptide inhibitor targeting SARS-CoV M^pro^. It exhibits a significant inhibitory effect with a K_i_ value of 0.035 µM, and an IC_50_ of 0.124 µM^61^. Its antiviral activity was investigated using a cytopathic effect (CPE) assay in Vero cells, exhibiting complete blockade of viral proliferation at a concentration of 10 μM. Notably, compound **27** (**YH-53**) demonstrated no cytotoxicity at concentrations up to 100 µM in Vero cells. This favourable cytotoxicity profile makes it a promising candidate for further therapeutic development. The inhibition mechanism involves the formation of a reversible hemi thioketal adduct via a reaction between the thiol group of Cys145 and the carbonyl carbon at the P1 position of the inhibitor as depicted by (PDB: 7E18)^60^. The ability of this promising inhibitor to bind to different M^pro^’s from different variants was also thoroughly investigated^62^. Through the elucidation of their crystal structures, compound **27** was found to bind successfully to all mutated M^pro^ structures and was also able to form the covalent bond with Cys145 in all investigated mutants (beta, lambda, delta, omicron). This indicates that compound **27** can retain its inhibitory activity against different strains of the rapidly mutating virus.

##### Fluor benzothiazole ketone

Compounds **28** (**TKB245**) and **29** (**TKB248**) are synthetic examples of peptidomimetics bearing a fluor-benzothiazole ketone warhead (Figure 8C). Compound **28** (**TKB245**) exhibits an IC_50_ value of 0.007 µM, making it an extremely potent inhibitor. In cell-based assays using VeroE6 cells, it shows an EC_50_ value of 0.03 µM, indicating strong antiviral activity^63^. Compound **28** (**TKB245)** maintains its efficacy across different SARS-CoV-2 strains, with EC_50_ values ranging from 0.014 to 0.056 µM, showcasing its robustness against viral mutations. Compound **29** (**TKB248**), which differs from **28** (**TKB245**) by having a thiocarbonyl group instead of a carbonyl group, has an IC_50_ value of 0.074 µM. In the same VeroE6 cell-based assays, it presents an EC_50_ value of 0.22 µM. While slightly less potent than **28** (**TKB245)**, **29** (**TKB248)** also retains significant antiviral activity against various SARS-CoV-2 strains, with EC_50_ values between 0.070 − 0.430 µM. The covalent bond formation is confirmed by crystallography (PDB: 8DOX and 8DPR, respectively)^63^. Both inhibitors had a CC_50_ > 100 µM in VeroE6, HeLa-ACE2-TMPRSS2, and A549-ACE2-TMPRSS2 cell lines.

#### Micheal acceptors

Michael acceptors encompass a diverse group of warheads that share the ability to undergo Michael addition reactions, forming irreversible or reversible covalent bonds with nucleophilic residues in proteins or other biomolecules^64^. Michael acceptors such as α, β-unsaturated carbonyl containing a conjugated carbon-carbon double bond, enables these compounds to act as electrophiles and react with nucleophiles, such as thiols (-SH) in cysteine residues of proteins, undergoing nucleophilic addition reactions, leading to the formation of the nearly irreversible covalent bond with the target. Vinyl esters and vinyl sulphones are prominent examples of Michael acceptors commonly employed as warheads in drug design and development. Other examples also include nitroalkenes.

##### Vinyl esters

**Figure 9.**
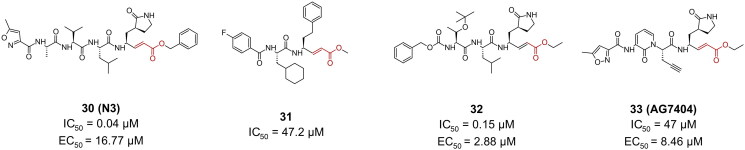
Chemical structures of peptidomimetic covalent inhibitors of SARS-CoV-2 M^pro^ with a vinyl ester (MA) warhead depicted in red. This figure was produced by the authors.

Compound **30** (**N3**) was initially developed via computer-aided drug design as an inhibitor of MERS-CoV and SARS-CoV proteases and was one of the first discovered inhibitors against SARS-CoV-2 (Figure 9). It contains a Michael acceptor warhead with a vinyl ester substructure that irreversibly modifies the Cys145 residue via its activated double bond. Cell-based assays conducted in Ver 6 cells, reported an EC_50_ value of 16.77 µM, and its crystal structure (PDB: 6LU7)^8^ revealed the expected covalent linkage between the compound’s C_β_ and Cys145^8^. It reported a CC_50_ of >133 µM in Vero cells. Compound **31** was among the top hits in a virtual screening campaign aiming at finding more potent **N3** analogues (Figure 9). Its IC_50_ was found to be moderate, at 47.2 µM, and the covalent bond formation was confirmed via dialysis assay^65^. No anti-viral cell-based assays were performed.

Compound **32** is a more potent analogue of compound **30** (**N3)** discovered via *in-vitro* screening, with an EC_50_ of 2.88 µM in Vero E6 cells and an IC_50_ of 0.15 µM^66^ (Figure 9). It exhibited no cytotoxicity at the tested concentration. Its crystal structure (PDB: 7JT7 and 7JW8)^66^ confirmed the covalent bond formation.

Compound **33** (**AG7404**) (Figure 9), originally developed as an orally bioavailable drug against human rhinovirus, has been repurposed in a study targeting the M^pro^ of SARS-CoV-1 and SARS-CoV-2^67^. **33** was found to exhibit an IC_50_ value of 47 μM against SARS-CoV-2 M^pro^, while demonstrating even greater inhibitory capacity against SARS-CoV-1 M^pro^, with an IC_50_ value of 29 μM. It reported an EC_50_ value of 6.80 µM in cell-based inhibition assays with A549-ACE2 cell lines and SARS-CoV-2, and a CC_50_ of >250 µM. Detailed analysis of protein-inhibitor interactions indicated that **33** fits well within the active site of the target proteases. This was further confirmed by crystallographic data, with the crystal structure (PDB: 7ZQV)^67^ revealing the formation of a covalent bond between **33** and Cys145 of the M^pro^.

##### Nitroalkenes

**Figure 10.**
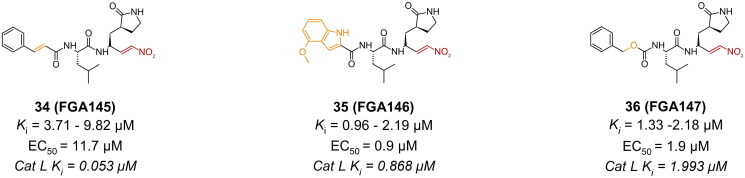
Chemical structures of peptidomimetic covalent inhibitors of SARS-CoV-2 M^pro^ with a nitroalkene (MA) warhead depicted in red, and first-point modification depicted in orange. This figure was produced by the authors.

A recent study reported three synthetic peptidyl nitroalkenes as promising SARS-CoV-2 M^pro^ inhibitors^68^. Compounds **34** (**FGA145**), **35** (**FGA146**), and **36** (**FGA147**) were synthesised based on previous work done on inhibitors of cysteine proteases (Figure 10). They have the common γ-lactam at the P1 position and leucine at the P2 position. All three compounds also have an aromatic ring at the P3 position. Compounds **35** (**FGA146**), and **36** (**FGA147**) were synthesised as analogues of compound **23** (**PF-00835231**), and compound **4** (**GC373**), respectively. Their tested inhibition activity reported K_i_ values ranging from 0.96 − 10 µM, retrieved from two assays with different M^pro^ expression vectors. These compounds were reported as reversible, as no irreversibility patterns were observed over a 10-min period. Compounds **35** (**FGA146**), and **36** (**FGA147**) reported an antiviral activity in Huh-7-ACE2 cells of 0.9 µM and 1.9 µM, respectively. Compound **34** (**FGA145**) on the other hand, had a higher EC_50_ of 11.7 µM. All three compounds reported a CC_50_ of >100 µM. Additionally, all three inhibitors reported impressive inhibition against cathepsin L at K_i_ values of 0.053 µM, 0.868 µM, and 1.993 µM, respectively, making them potential multi-target inhibitors and useful as antiviral agents. The crystal structures of compounds **35** (**FGA146**) (PDB: 8BGA)^68^, and **36** (**FGA147**) (PDB: 8BGD)^68^ confirm the formation of a covalent bond with Cys145 through the C19 of the nitroalkene via Micheal addition.

#### α-ketoamides

The α-ketoamides form reversible covalent bonds and the resulting -OH group serves as a hydrogen bond donor, forming an additional stabilising H-bond with His41^69^. Furthermore, the oxygen atom within the carboxamide group of α-ketoamides can engage in H-bond interactions with neighbouring backbone amide groups, such as those from Gly143, Ser144, and Cys145^69^. This collective interaction profile contributes to the enhanced stability and binding affinity of these ligands within the enzyme’s active site.

**Figure 11.**
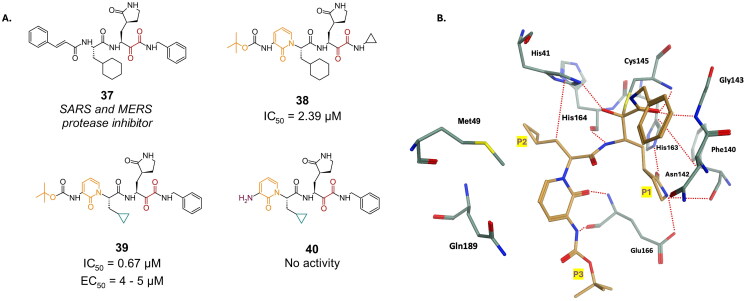
**A.** Chemical structures of peptidomimetic covalent inhibitors of SARS-CoV-2 M^pro^ with an α-ketoamide warhead depicted in red, first-point modification depicted in orange, second-point modification depicted in blue, and third-point modification depicted in purple. **B.** X-ray crystal structure of 6Y2F, depicting the fitting and interactions of ligand **39** in the M^pro^’s active site. This figure was produced by the authors.

Compound **37** was one of the early structures investigated within this warhead category (Figure 11A). Originally designed as an inhibitor for MERS and SARS-CoV, it demonstrated acceptable inhibition against the proteases of both viruses, with potency in the picomolar and micromolar range, respectively. Utilising a FRET enzyme-based assay, compound **37** exhibited an IC_50_ of 0.18 µM against the M^pro^ enzyme of SARS-CoV-2^10^^,^^70^. Subsequent optimisation of compound **37** led to the development of compound **38** (Figure 11A), which underwent modifications at the P2 and P3 positions by constraining them into a pyridine ring^10^. Additionally, to enhance cell permeability by reducing the molecule’s plasma protein binding affinity, the cinnamoyl linker was replaced with a less bulky tert-butoxy carbonyl (Boc) group. These modifications resulted in an IC_50_ value of 2.39 µM. Further refinements at the P2 position involved replacing the cyclohexyl moiety with a cyclopropyl group, yielding compound **39** (Figure 11A), with improved IC_50_ of 0.67 µM, and an EC_50_ 4–5 µM is observed in human Calu3 cells. The crystal structure of **39** (PDB: 6Y2F)^10^ (Figure 11B) reveals the formation of a covalent bond between the α-keto group and the Cys145 residue, yielding a reversible thioemiketal adduct. Much like the case with peptidyl aldehydes discussed in the previous section, the P1 position occupied by a Gln surrogate, is accommodated in the S1 sub-pocket. In the case of α-ketoamides, the P1 position houses a γ-lactam, fostering bifurcated H-bonds with Phe140, Glu166, and His163. In an attempt to further simplify the structure via elimination, the Boc group was removed yielding compound **40** (Figure 11A), which showed no activity against the SARS-CoV-2 M^pro^ enzyme.

**Figure 12.**
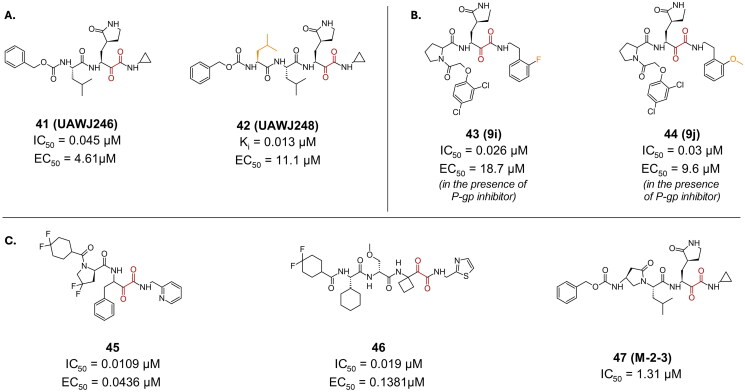
Chemical structures of **A.** GC376 peptidomimetic derivatives, **B.** di-peptide based inhibitors, and **C.** other examples of peptidomimetic covalent inhibitors of SARS-CoV-2 M^pro^ with a ketoamide warhead depicted in red, and first-point modification depicted in orange. This figure was produced by the authors.

Compounds **41** (**UAWJ246**) and **42** (**UAWJ248**) (Figure 12A) were developed using compound **4’s** (**GC373**) scaffold as a foundation^43^. However, their *in-vitro* testing and cell-based assays in Vero E6 cells indicated that substituting the aldehydic warhead with a ketoamide did not result in any discernible enhancement in inhibitory or antiviral properties, with compound **41** exhibiting an IC_50_ of 0.045 µM and an EC_50_ of 4.61 µM. Their CC_50_ was >100 µM in multiple investigated cell lines (Caco-2, HCT-8, Huh-7, MDCK, and Vero cell lines).

Compounds **43** (**9i**) and **44** (**9j**) are dipeptide-based inhibitors designed with an α-ketoamide warhead, featuring a γ-lactam group at the P1 position and a proline residue at the P2 position (Figure 12B)^71^. These compounds have demonstrated highly potent inhibitory effects against the SARS-CoV-2 M^pro^, with IC_50_ values of 0.026 µM for compound **43** and 0.03 µM for compound **44**. Both compounds were reported as inactive when investigated for their antiviral activity in Vero E6 cells (EC_50_ > 100 µM). However, it was noted that in the presence of a P-gp inhibitor, the EC_50_ values reported are 18.7 and 9.6 µM for compounds **43** and **44**, respectively. Their reports CC_50_ was >100 µM. Additionally, both compounds have been investigated for their inhibitory activity against the MERS-CoV protease, showing IC_50_ values of 20.3 µM and 24.6 µM for compounds **43** and **44**, respectively. These results suggest that these compounds possess the potential to act as broad-spectrum antivirals, or pan-coronavirus inhibitors, that can effectively neutralise infections caused by different strains of this virus.

Through SAR studies on a series of synthesised α-ketoamide derivatives, compound **45** was synthesised, and reported as a potent M^pro^ inhibitor with an IC_50_ value of 0.0109 µM (Figure 12C)^72^. The covalent bond formation was confirmed via crystallography (PDB: 8I30)^72^. In *in-vitro* antiviral assay, it reported an EC_50_ value of 0.0436 µM, and a CC_50_ of >100 µM in Vero E6 cells.

α-ketoamides, such as those found in drugs like Boceprevir, often face the challenge of epimerization under physiological conditions, which can convert them into inactive configurations, thus compromising their efficacy. In an effort to address the epimerization challenge, researchers have developed derivatives incorporating quaternized P1 groups^73^. This resulted in compound **46**, which has shown potent enzymatic inhibitory activity against SARS-CoV-2 M^pro^, with an IC_50_ value of 0.019 µM (Figure 12C). Additionally, it exhibited excellent antiviral activity in cell-based assays, with an EC_50_ value of 0.1381 µM in HPAEpiC cells.

In a recent study, researchers explored a novel scaffold of α, γ-AA peptides as potential inhibitors of SARS-CoV-2 M^pro^^74^. They synthesised a new class of peptidomimetics-constrained α, γ-AA peptides, inspired by a series of aldehyde and ketoamide inhibitors targeting the M^pro^ enzyme. Amongst these, Compound **47** (**M-2–3**), featuring a ketoamide warhead, emerged as a promising hit with an IC_50_ value of 1.31 µM, and a CC_50_ > 60 µM in Vero E6 cells (Figure 12C). Its antiviral activity was however not investigated is cell-based assay. Covalent bond formation was confirmed via crystallography (PDB: 8DZB)^74^.

**Figure 13.**
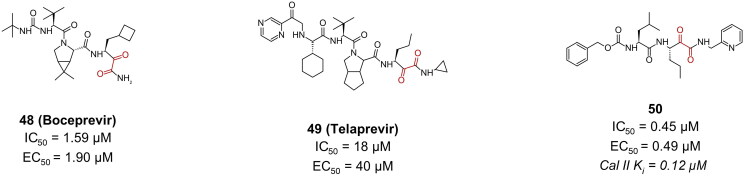
Chemical structures of peptidomimetic covalent inhibitors of SARS-CoV-2 M^pro^ with a ketoamide warhead depicted in red. This figure was produced by the authors.

Compounds **48** (boceprevir) and **49** (telaprevir), which are FDA approved hepatitis C antivirals, exemplify efforts in drug repurposing against the SARS-CoV-2 M^pro^ (Figure 13). Their crystal structures (PDB: 6ZRU and 6ZRT, respectively)^75^ confirm the covalent bond formation. Compound **49** exhibited a relatively high IC_50_ of 18 µM^76^, and an EC_50_ of 40 µM, along with a CC_50_ > 432 µM in Vero E6 cells^77^. In contrast, compound **48** displayed a more promising profile, boasting an IC_50_ of 1.59 µM and an EC_50_ of 1.90 µM^75^. Additionally, it demonstrated a safer profile, with a CC_50_ > 100 µM in Caco-2 cells.

Compound **50** (Figure 13) is an example of a tripeptidyl α-ketoamide calpain II inhibitor (K_i_ = 0.12 µM)^78^, reporting a rather impressive antiviral activity in Vero E6 cells, with an EC_50_ value of 0.49 µM, and an inhibitory activity (IC_50_) of 0.45 µM^50^. Its CC_50_ was >100 µM. The remarkable efficacy associated with calpain inhibitors against the M^pro^ enzyme can be explained via their “dual-target” nature. As calpain II and cathepsin L are key enzymes playing a crucial role in the viral penetration of host cells via processing the viral S protein, simultaneous inhibition of both enzymes could lead to a superior antiviral activity, meanwhile reduce the risk of resistance.

#### Halo-acetamide

Alkyl halogens at the α carbonyl position under this category are known to form an irreversible covalent bond following the reaction between the warhead and the thiolate of Cys145.

##### α-mono halo acetamide

**Figure 14.**
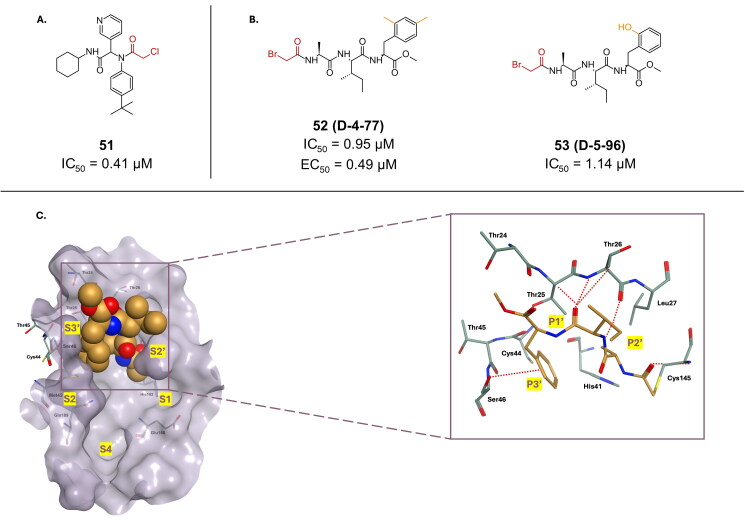
Chemical structures of peptidomimetic covalent inhibitors of SARS-CoV-2 M^pro^ with an α-halo acetamide warhead depicted in red from **A.** *in-vitro* sources and **B.** synthetic sources. **C.** X-ray crystal structure of 8JPQ, depicting the fitting and interactions of ligand **50** in the M^pro^’s active site in S1′ to S3′ sub-pockets. This figure was produced by the authors.

Compound **51** bearing an α-chloro acetamide, was reported as the most promising lead in an *in-vitro* study targeting the SARS-CoV-2 M^pro^ enzyme with an IC_50_ of 0.41 µM (Figure 14A)^79^. The crystal structure (PDB: 7MLF)^79^ reveals a displacement of the chloride in favour of covalent bond forming thiolate of Cys145 in the active site. Its antiviral activity was however not investigated.

In a study aimed at investigating the significance of the S1′, S2′, and S3′ subsites in the inhibition of SARS-CoV-2 M^pro^, researchers employed rational drug design to develop a series of potential inhibitors^80^. Compounds **52** (**D-4–77**) and **53** (**D-5–96**), which are peptidyl inhibitor featuring an α-bromo acetamide warhead (Figure 14B), was synthesised as part of this effort, exhibiting a promising inhibition profile, with an IC_50_ value of 0.95 μM and 1.14 µM, respectively. The antiviral profile of **52** was also found to be promising, with EC_50_ value of 0.49 μM, with no apparent cytotoxicity towards Vero E6 cells. The inhibitory mechanism of compound **53** was further elucidated through the co-crystal structure (PDB: 8JPQ)^80^, confirming that the inhibitor binds covalently to the catalytic site of M^pro^, specifically engaging the S1′–S3′ subsites (Figure 14C). The α-bromo acetamide warhead forms a covalent bond with Cys145 in M^pro^, stabilising the inhibitor within the active site and effectively blocking the protease’s function. The carbonyl oxygen of α-bromo acetamide forms a hydrogen bond with the amidic group of Cys145. The Ala at P1′ interacted with Thr25, Thr26, and Leu27 via van der Waals interactions. The amidic group and carbonyl oxygen of Ile (P2′) formed hydrogen bonds with the carbonyl oxygen and amidic group of Thr26, respectively. The Phe (P3′) residue occupied the S3′ pocket formed by Cys44, Thr45, Met49. These interactions allowed the inhibitor to bind to the S1′–S3′ pocket and facilitated the covalent attachment of the warhead to Cys145.

##### α-di halo acetamide and α-tri halo acetamide

**Figure 15.**
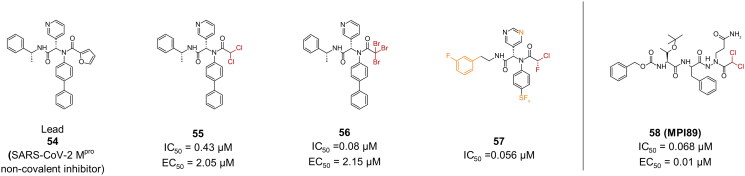
Chemical structures of peptidomimetic covalent inhibitors of SARS-CoV-2 M^pro^ with an α-di and tri-halo acetamide warhead depicted in red, and first-point modification depicted in orange. This figure was produced by the authors.

Making use of compound **54**, a noncovalent inhibitor of SARS-CoV-2 M^pro^ enzyme, a number of peptidomimetic inhibitors with halo acetamide warheads were designed, by replacing the furan ring with a di or tri-halo acetamide warhead (Figure 15)^81^. The most promising leads, compounds **55**, and **56**, carry 2,2 di chloro, and tri bromo warheads, respectively. While compound **56** has a more promising IC_50_ of 0.08 µM compared to **55** (IC_50_ = 0.43), they both report comparable impressive EC_50_ values, at 2.15 µM and 2.05 µM, respectively. However, compound **55** exhibited a safer profile in Vero cells with a CC_50_ > 100 µM compared to 5.48 µM in the case of compound **56**. The crystal structure of compound **55** (PDB: 7RN1)^81^ confirmed the covalent bond formation and emphasised the importance of the R configuration for the pyridyl, to allow for insertion into the S1 site.

Encouraged by the results and potency of the previous compounds, compounds **55** and **56** were utilised as lead compounds, producing compound **57**, with an interesting α-chloro-fluoroacetamide warhead, and a substantially improved IC_50_ of 0.056 µM (Figure 15)^82^. The antiviral activity was however not investigated. The covalent bond formed by this warhead appears to be pseudo reversible, meaning that off-target activity could greatly be minimised.

In a recent study, compound **58** (**MPI89**), an inhibitor featuring an aza-2,2-dichloroacetyl warhead (Figure 15), was successfully synthesised alongside a diverse array of azapeptide inhibitors, each designed with various covalent warheads targeting the catalytic cysteine of SARS-CoV-2 M^pro^^83^. Compound **58** demonstrated a remarkable EC_50_ value of 0.01 µM against SARS-CoV-2 infection in ACE2 cells, underscoring its potent antiviral activity. It exhibits no activity against SARS-CoV-2 papain-like protease (PL^pro^) or several human proteases (transmembrane protease serine 2 (TMPRSS2), furin, and cysteine proteases cathepsins B/K/L), which highlights its specificity for M^pro^, which is crucial for minimising off-target effects and potential side effects in therapeutic applications. Additionally, it exhibited a CC_50_ of 59.6 µM of in HEK293T cells. Crystallography analyses revealed a covalent bond formation with Cys145 (PDB: 8S9Z)^84^.

#### Nitriles

**Figure 16.**
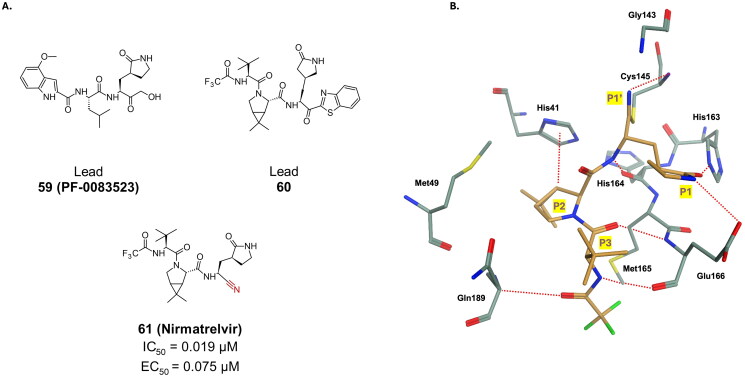
**A.** Chemical structures of peptidomimetic covalent inhibitors of SARS-CoV-2 M^pro^ with a nitrile warhead depicted in red. **B.** X-ray crystal structure of 7VH8, depicting interactions of ligand **61** in the M^pro^’s active site. This figure was produced by the authors.

Nitriles are reversible inhibitors of M^pro^ as they form a transient thioimidate bond with Cys145. Despite their moderate efficacy as coronavirus inhibitors in earlier studies^85^, nitriles remain useful in developing orally available drugs by reducing hydrogen bond donors^86^, thereby enhancing the drug’s permeability^86^. Pfizer utilised this approach to optimise compound **59** (P**F-0083523**), an α-hydroxymethyl ketone, as well as compound **60** and developed compound **61** (**nirmatrelvir/PF-07321332**) (Figure 16A) reporting exceptional inhibitory and antiviral activity against SARS-CoV-2 M^pro^ (IC_50_ = of 0.019 µM, EC_50_ = 0.075 µM in Vero E6 cells)^87^. The X-ray crystal structure (PDB: 7VH8^88^ and 7VLQ^89^) of compound **61** reveals the formation of a thioimidate with Cys145, stabilised by a H-bonding between the imine and Gly143, (Figure 16B)^88^. It also showed no cytotoxicity in cell-based assays. Clinical trials qualified compound **61** for emergency approval by the FDA in December 2021. Co-administration with ritonavir improved drug performance by inhibiting CYP-mediated metabolism, thereby increasing the drug half-life, leading to co-formulation with nirmatrelvir in the final drug product, paxlovid^86^.

Several studies have documented the effectiveness of compound **61** against various strains of the virus, including beta, delta, and omicron^90–92^. This is particularly encouraging considering the coronavirus’s propensity for a high mutation rate, which poses challenges in containing its spread through other measures, such as vaccines. Results from clinical trials reveal a favourable pharmacokinetics profile, as well as persistent viral clearance, and reassuring drug safety profile in healthy subjects^93^. A dose reduction was advised at the end of another study focusing on renally impaired individuals, due to the drug’s increased plasma concentration^94^.

**Figure 17.**
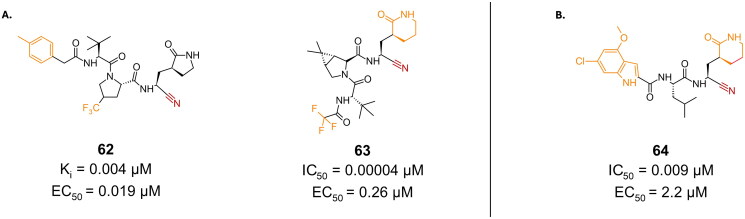
Chemical structures of **A.** nirmatrelvir and **B.** GC376 peptidomimetic derivatives as covalent inhibitors of SARS-CoV-2 M^pro^ with a nitrile warhead depicted in red, and first-point modifications depicted in orange. This figure was produced by the authors.

Through alteration on the P2 and P3 position, Pfizer patented compound **62**, which is another promising candidate bearing a nitrile warhead, and reporting strong binding against the M^pro^ enzyme, with a K_i_ of 0.004 µM and a superior antiviral activity with an EC_50_ value of 0.019 µM (Figure 17A)^95^. Compound **63** is a synthetic derivative of **61** (**nirmatrelvir**), modified at the P4 position (Figure 17A)^96^. The 6-membered lactam at P4 led to more than 6-fold improvement of M^pro^ inhibitory activity (K_i_ = 0.04 nM) as well as 7-fold improvement in antiviral activity (EC_50_ = 0.26 µM), in Vero E6 cells, compared to the in-house synthesised nirmatrelvir (K_i_ = 0.26 µM, and EC_50_ = 2.0 µM). Covalent bond formation was confirmed through crystallography (PDB: 8UND)^97^.

Compound **64** is another example of nitrile bearing inhibitors, designed by a research group that used compound **4** (**GC376**) and it derivatives as their lead (Figure 17B)^98^. Their most promising candidate (compound **64**) reported an IC_50_ of 0.009 µM and EC_50_ value of 2.2 µM in Vero E6 cells, as well as a CC_50_ of >200 µM.

#### Latent alkyne

**Figure 18.**
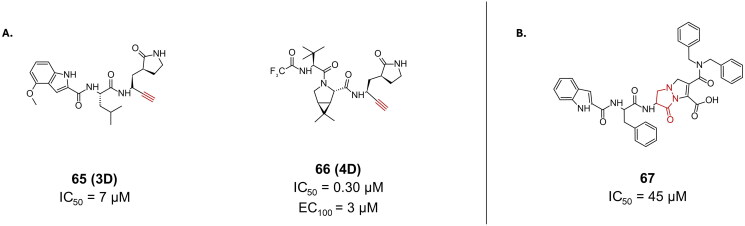
Chemical structures of peptidomimetic covalent inhibitors of SARS-CoV-2 M^pro^ with **A.** an alkyne and **B.** a pyrazolidinone warhead, depicted in red. This figure was produced by the authors.

Building on scaffolds of known M^pro^ inhibitors that are clinically used or underwent clinical evaluations, one research group used a panel of peptidomimetics with varying warheads, such as esters, vinyl sulphones, terminal alkynes, aldehydes, ketoamides, and nitriles^99^, landing on terminal alkynes as potential warheads that covalently bind to Cys145 in the M^pro^ of SARS-CoV-2. Terminal alkynes portrayed a latent reactivity, that can be activated in the active site due to catalytic environment. This is thought to be rather beneficial, as it acts as an elegant solution towards the off-target risk associated with the use of irreversible covalent inhibitors, due to the fact that latent warheads lack intrinsic reactivity. Compounds **65** (**3D**) and **66** (**4D**) were among the most potent derivatives bearing a terminal alkyne warhead with IC_50_ values of 7 µM and 0.30 µM, respectively, (Figure 18A). These compounds have the same structure as compound **23** (**PF-00835231**) and compound **61** (**Nirmatrelvir**), but their respective warheads have been changed to terminal alkyne. This class of warhead is suspected to result in an irreversible covalent bond, forming a vinyl thioether linkage, leading to the inactivation of the target. This was confirmed by the time-dependent inhibition assay, followed by MS analysis to confirm that the reaction occurs with Cys145. Crystal structures of both compounds (PDB: 8FY7 and 8FY6, respectively)^99^ confirmed the covalent bond formation with the internal carbon of the alkyne. Furthermore, the dose-dependent antiviral activity of compound **66** was rather promising, reporting almost complete inhibition at 3 µM in HeLa-ACE2 cells. It also reported no notable cytotoxicity up to 10 µM (the highest tested concentration).

#### Pyrazolidinone

One recent study investigating β- and **γ**-lactams as electrophilic “warheads” for covalent binding to Cys145 of the M^pro^ active site, reported compound **67**, a dipeptide with a pyrazolidinone warhead (Figure 18B)^100^. It reported the highest inhibitory activity with an IC_50_ value of 45 μM, among all other tested compounds, but its antiviral activity was not investigated. The inhibition mechanism was confirmed by 2D-NMR, revealing that the covalent bond formation occurs as a result of **γ**-lactam ring opening of the pyrazolidinone.

Reflecting on the various cases discussed, it is noteworthy that computer-aided drug design was not employed in the discovery of the most effective peptidomimetic inhibitors. While computer-aided drug design has been instrumental in identifying both non-peptidomimetic (discussed in the next section) and non-covalent inhibitors against SARS-CoV-2 M^pro^, its triumphs predominantly lie in the realm of drug repurposing and the optimisation/derivatization of existing compounds. These efforts have successfully enhanced the activity of known inhibitors, steering the development towards non-peptidomimetic, covalent inhibitors. However, the application of computer-aided drug design in discovering novel peptidomimetic scaffolds for the development of covalent inhibitors appears somewhat limited.

### Non-peptidomimetics inhibitors

Although numerous peptidomimetic inhibitors with varying success have been reported, exploring non-peptidomimetic leads have gained interest as it offers a valuable strategy for diversifying the chemical space of potential inhibitors and discovering novel therapeutics with unique mechanisms of action and improved properties. Table 2 depicts some of the potent warheads found in non-peptidomimetic compounds. The most notable examples will be discussed in the following sections.

**Table 2.** Electrophilic warheads found in current non-peptidomimetic M^pro^ inhibitors.

Electrophilic warhead	Mechanism	Structure
Vinyl sulphonamide	Michael Addition	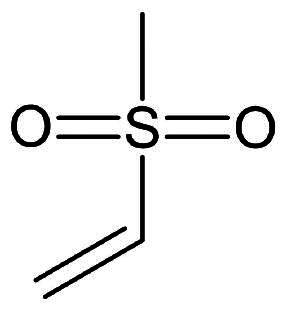
Ester	Michael Addition	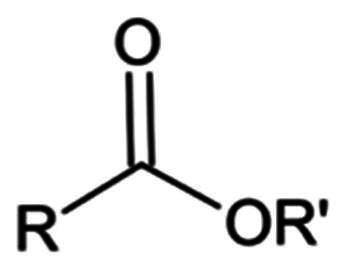
2-Haloactamide	Nucleophilic Substitution	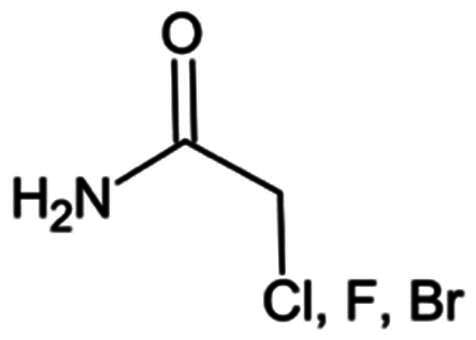
Acrylamide	Michael Addition	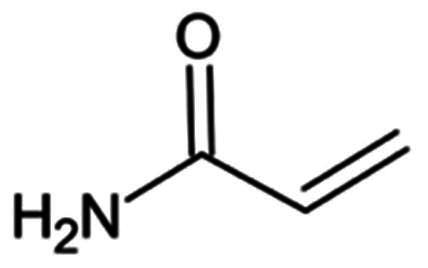
Ketone	Nucleophilic Addition to a Double Bond	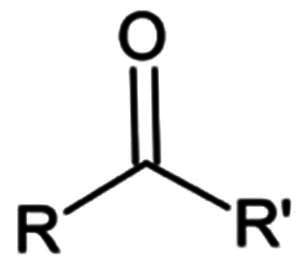
Nitrile	Nucleophilic Addition to a Triple Bond	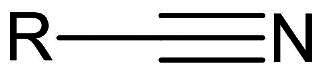
Epoxide	Nucleophilic substitution (SN2)	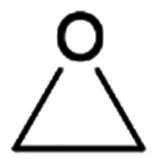
Michael Acceptor	Michael Addition	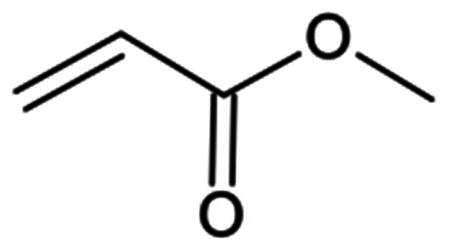

*Abbreviations:* M^pro^, main protease; PL^pro^, papain-like protease; COVID-19, Coronavirus Disease 2019; WHO: World Health Organisation; CFR, case fatality rate; H-bond, hydrogen bond; IC_50_, a half-maximal inhibitory concentration; EC_50_, half maximal effective concentration; CC_50_, cytotoxicity concentration; FCoV, feline coronavirus; FIP, fatal feline infectious peritonitis; MA, Micheal.

#### Non-peptidomimetic inhibitors from synthetic routes

Synthetic non-peptidomimetic inhibitors of SARS-CoV-2 M^pro^ have garnered significant attention in the search for potential therapeutics against COVID-19. Some of the most notable examples are discussed below.

##### Vinyl sulphone

**Figure 19.**
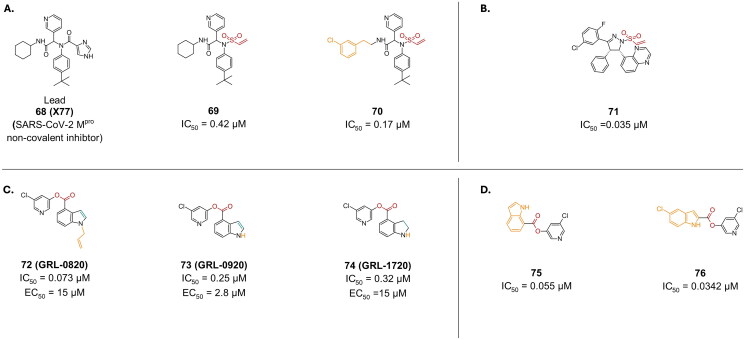
Chemical structures of non-peptidomimetics from synthetic routes as covalent inhibitors of SARS-CoV-2 M^pro^ with **A.** and **B.** a vinyl sulphone and **C.** and **D.** an ester warhead, depicted in red, first-point modifications depicted in orange, and second-point modification depicted in blue. This figure was produced by the authors.

Vinyl sulphones are known for forming covalent bonds with the cystine protease in an irreversible manner, via their β-vinyl carbon. Compound **68** (**X77**) is an example of non-covalent SARS-CoV-2 M^pro^ inhibitor, that was utilised to design the non-peptidomimetic compounds **69** and **70**, with a vinyl sulphone warhead instead of the imidazole moiety in **68** (Figure 19A)^79^. Derivative **69** reported a promising IC_50_ value of 0.42 µM, which is impressive considering the IC_50_ of the parent compound (4.1 µM). The covalent bond formation was confirmed by crystallography (PDB: 7MLG)^79^. Replacing the cyclohexyl at P3 to the longer 3-chlorophenyleth-2-yl group produced the more potent compound **70**, with an improved IC_50_ of 0.17 µM. Another example is compound **71**, with a pyrazoline skeleton and a promising IC_50_ (0.035 µM) (Figure 19B)^101^. Interestingly, this compound also shows activity against other coronaviruses, such as SARS-CoV, HCoV-NL63, IBV, and HCoV-229E. However, due to their solubility issues, metabolic stability, and cell permeability, their antiviral activity was not investigated in cells.

##### Esters

Another group of SARS-CoV-2 inhibitors acting via the formation of an irreversible bond is the ester. The leaving group is eliminated upon the attack by the nucleophilic thiolate, resulting in acylation of the target irreversibly. The importance and effectiveness of small M^pro^ inhibitors were highlighted through a series of indole/indoline chloropyridinyl previously reported as active inhibitors of SARS-CoV M^pro^ emerging as promising inhibitors of SARS-CoV-2 M^pro^. Compounds **72** (**GRL-0820**), **73** (**GRL-0920**), and **74** (**GRL-1720**) were among the most potent leads, with IC_50_ values of 0.073 µM, 0.25 µM, and 0.32 µM, respectively, (Figure 19C)^102–104^. Compound **73** (**GRL-0920)** also exhibited a rather impressive antiviral activity in Vero E6 cells, with an EC_50_ value of 2.8, while compounds **72** (**GRL-0820**) and **74** (**GRL-1720**) had a more modest EC_50_ of 15 µM. Compound 74 reported a CC_50_ value of >100 µM in Vero E6 cells. The antiviral activity of compound’s 72 and 73 were confirmed using immunocytochemistry assays in Vero E6 cells. Another study reported compounds **75** and **76,** which are also chloro-substituted pyridinyl indole esters, as the most potent, with IC_50_ values of 0.055 µM and 0.0342 µM, respectively, **(**Figure 19D)^105^.

##### α-halo acetamide

**Figure 20.**
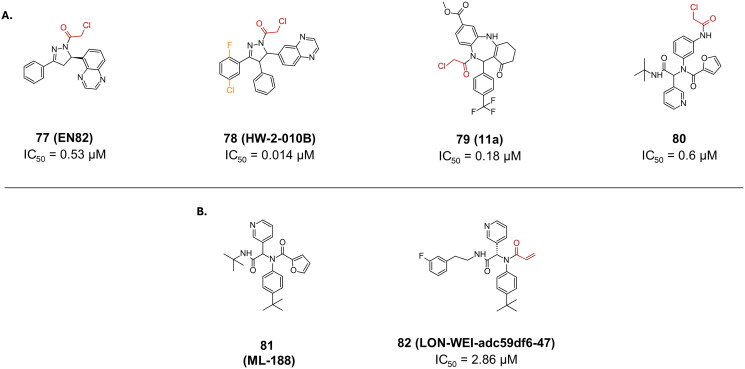
Chemical structures of non-peptidomimetics from synthetic routes as covalent inhibitors of SARS-CoV-2 M^pro^ with **A.** an α-Halo acetamide and **B.** an acrylamide warhead, depicted in red, and first-point modification depicted in orange. This figure was produced by the authors.

Pyrazoline-based scaffolds were also investigated via activity-based protein profiling, and compound **77** (**EN82**) was reported as a potent M^pro^ inhibitor with an IC_50_ of 0.53 µM (Figure 20A). Through SAR studies, compound **78** (**HW-2010B**) was derivatized reporting an improved inhibition in the nanomolar range (0.014 µM) (Figure 20A)^101^. However, cell-based assays are still needed to investigate their anti-viral activity, and possible cytotoxicity.

Through SAR studies and derivatization efforts, compound **79** (**11a**), a benzodiazepine derivative with a chloroacetamide was identified as a potent M^pro^ inhibitor with an IC_50_ value of 0.180 μM (Figure 20A)^106^. Crystallography confirmed covalent bond formation (PDB: 8JOP)^106^.

Compound **80** with a chloroacetamide was reported as a potent SARS-CoV-2 M^pro^ inhibitor, with a promising IC_50_ of 0.6 μM (Figure 20A)^107^. However, it reported a CC_50_ of 12.1 µM in Vero cells. Crystallography confirmed covalent bond formation (PDB: 8TPB)^107^.

##### Acrylamide

Acrylamides are another example of cysteine inhibitors acting via covalent bond formation that is in most cases irreversible. One study started from compound **81** (**ML188**), a non-covalent SARS-CoV/SARS-CoV-2 inhibitor, to develop compound **82** (**LON-WEI-adc59df6-47**), a non-peptidomimetic covalent inhibitor of M^pro^ (Figure 20B)^108^. Only the S conformation was reported to be active, with an IC_50_ value of 2.86 µM. Its crystal structure (7NW2) reveals a covalent bond between the Cys145 thiolate and the β-vinyl carbon of the acrylamide moiety replacing the furan ring in the parent structure. Additionally, the para tertbutyl phenyl moiety is accommodated within the S2 hydrophobic sub pocket. Finally, the newly introduced phenylethylamide moiety occupies the S4 cleft, resulting in key interactions with surrounding residues.

##### Nitriles

**Figure 21.**
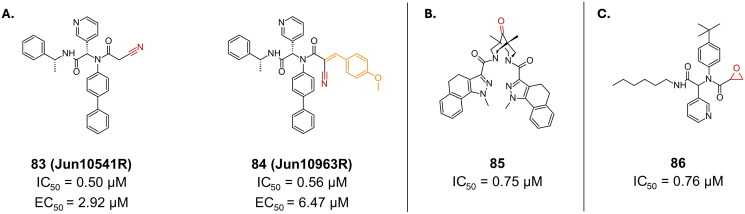
Chemical structures of non-peptidomimetics from synthetic routes as covalent inhibitors of SARS-CoV-2 M^pro^ with **A. a** nitrile, **B.** a ketone and **C.** an epoxide warhead depicted in red, and first-point modification depicted in orange. This figure was produced by the authors.

A recent study set out to explore various electrophilic warheads, as potential inhibitor of the SARS-CoV-2 M^pro^ enzyme^109^. Some of their most potent hits carried a nitrile warhead. Compounds **83** (**Jun10541R**), and **84** (**Jun10963R**) specifically carrying a nitrile warhead, reported promising IC_50_ value of 0.50 µM, and 0.56 µM, respectively, (Figure 21A). They reported EC_50_ values of 2.92 µM and 6.47 µM in Calu-3 cell. They both had CC_50_ > 100 µM in E6 cells. The covalent bond formation was confirmed by the crystal structure of **83** (PDB: 8FIV)^109^.

##### Ketone

Compound **85** is a unique example of a non-peptidomimetic SARS-CoV-2 M^pro^ inhibitor, with a ketone warhead (Figure 21B)^110^. Its scaffold made up of bispidine exhibits a rather promising inhibitory activity with an IC_50_ of 0.75 µM. It was found to be non-toxic in HEK293T cell line.

##### Epoxide

Compound **86**, featuring an epoxide functional group, has been identified as a potent inhibitor of SARS-CoV-2 M^pro^, demonstrating an IC_50_ value of 0.76 μM (Figure 21C)^107^. The structural details of its inhibition mechanism were elucidated through crystallographic studies (PDB: 8TPF)^107^, revealing that the inhibition mechanism involves an SN2 substitution reaction between the electrophilic epoxy group and Cys145. Interestingly, the study highlighted an unusual aspect of this interaction: Cys145 attacks the more sterically hindered epoxy Cα atom rather than the less hindered Cβ atom, which would typically be more favourable for an SN2 reaction. This counterintuitive behaviour suggests that the spatial orientation and electronic properties of the active site facilitate this specific interaction.

#### Non-peptidomimetic inhibitors from random screening and repurposing approaches

By using *in-vitro* screening campaigns, researchers have identified molecules that show promising inhibitory activity against SARS-CoV-2 M^pro^, that are also non-peptidomimetic in nature. Repurposing existing non-peptidomimetic drugs has also yielded potent covalent inhibitors of SARS-CoV-2 M^pro^. This section discusses some of the most prominent examples.

##### Michael acceptors

**Figure 22.**
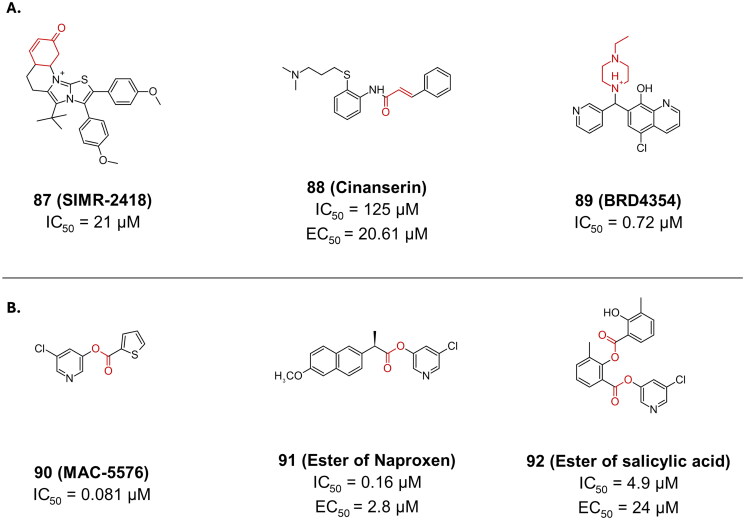
Chemical structures of non-peptidomimetics from synthetic routes as covalent inhibitors of SARS-CoV-2 M^pro^ with **A.** a Micheal acceptor, and **B.** an ester warhead depicted in red. This figure was produced by the authors.

An example of non-peptidomimetics bearing a Michael acceptor warhead, is compound **87** (**SIMR-2418**), featuring a fused benzo [b] [1,4] oxazin-6(5H) one-imidazo [2,1-b] thiazole scaffold (Figure 22A)^111^. It was reported as one of the most active hits with an IC_50_ of 20.7 µM, at the end of an *in-vitro* screening campaign. The fused system was suggested to be a key feature required for the appropriate orientation of the inhibitor in the M^pro^’s active site. The covalent inhibition on the other hand is possible due to the presence of the cyclohexanedione moiety.

Compound **88** (**cinanserin**) was identified in the same study that reported compound **30** (**N3**) as a promising SARS-CoV-2 M^pro^ inhibitor (Figure 22A)^8^. Originally reported as a 5-hydroxytryptamine receptor antagonist, use of **88** against M^pro^ is another example of drug repurposing approach utilised to find potent cysteine protease inhibitors for SARS-CoV-2. The structure of **88** bears an α, β-unsaturated carbonyl moiety, indicating a similar interaction pattern with the Cys145 through covalent bond formation. Its reported IC_50_ was rather high at 125 µM, while it EC_50_ was 20.61 µM, and it CC_50_ was >200 µM in Ver E6 cells.

In a study aimed at identifying novel covalent inhibitors of SARS-CoV-2 M^pro^, researchers screened a small library of metal-binding pharmacophores (MBPs)^112^. One of the standout compounds from this screening was compound **89** (**BRD4354**), which demonstrated potent inhibitory activity with an IC_50_ value of 0.72 μM (Figure 22A). The inhibition mechanism of **89** was investigated using MS analysis, which revealed that the compound’s thiol-reactive ortho-quinone methide covalently binds Cys145. This covalent bond formation effectively inactivates the enzyme. The proposed mechanism for this reaction involves a retro-Michael reaction followed by a Michael addition. In this process, the ortho-quinone methide undergoes a retro-Michael reaction to generate a reactive intermediate, which then participates in a Michael addition with the thiol group of Cys145. This sequence of reactions ensures the formation of a stable covalent bond, thereby inhibiting the protease’s activity.

##### Esters

Compound **90** (**MAC-5576**) is another example of a non-peptidomimetic ester that was previously reported as a SARS-CoV M^pro^ inhibitor (Figure 22B). It reported an inhibitory activity against the M^pro^ of SARS-CoV-2 of 0.081 µM^66^. Regardless of its promising IC_50_, its antiviral activity did not match once tested in Vero E6 cells, failing to block viral infection. However, it did not report any cytotoxicity at the tested concentrations. The covalent bond formation was confirmed via crystallography (PDB: 7JT0)^66^.

A series of 5-chloropyridinyl esters of nonsteroidal anti-inflammatory drugs were tested against the M^pro^ of SARS-CoV-2, reporting compounds **91** (5-chloropyridinyl ester of naproxen) and **92** (ester of salicylic acid) as promising (Figure 22B)^113^. Compound **91** had an encouraging IC_50_ of 0.16 µM, and an EC_50_ of 2.8 µM in Vero E6 cells, while compound **92** had a higher IC_50_ of 4.9 µM and an EC_50_ of 24 µM. Both compounds reported a CC_50_ of >100 µM.

##### Other examples

**Figure 23.**
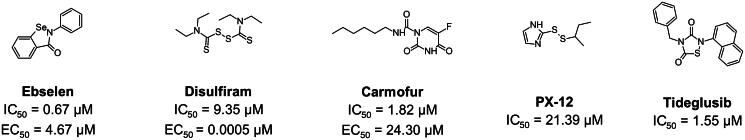
Examples of non-specific binders identified as SARS-CoV-2 M^pro^ covalent inhibitors. This figure was produced by the authors.

During the very beginning of the pandemic, a number of approved drugs, as well as drug candidate under clinical trials were identified via high-throughput screening as potential M^pro^ inhibitors. These include **ebselen**, **disulphiram**, **carmofur**, **PX-12**, and **Tideglusib** (Figure 23)^8^. They had rather promising IC_50_ values, ranging from 0.67 µM − 21.39 µM. Their ability to form covalent bonds was confirmed by MS analysis. However, very early on, these compounds were found to be non-specific and promiscuous in their inhibition profile^114^. Regardless of this finding, optimisation campaigns of these compounds to find more potent inhibitors are still ongoing.

#### Non-peptidomimetic inhibitors from computer-aided drug design

Several non-peptidomimetic compounds were identified as potential covalent inhibitors of the SARS-CoV-2 M^pro^, through computer-aided drug design. These studies utilised molecular docking, molecular dynamics simulations, and various other *in-silico* methodologies to identify and optimise inhibitors.

##### Ketone

**Figure 24.**
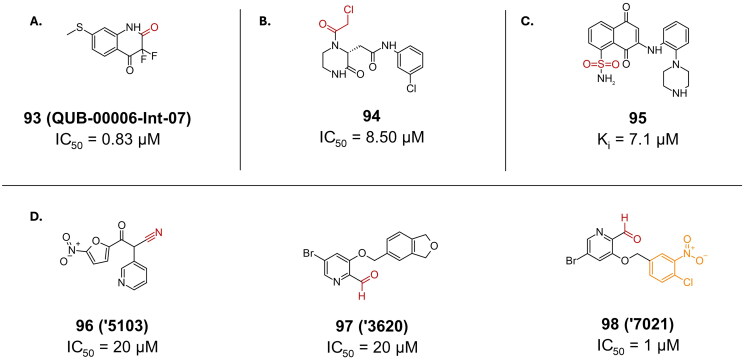
Chemical structures of non-peptidomimetics from computer-aided drug design as covalent inhibitors of SARS-CoV-2 M^pro^ with **A.** a ketone, **B.** a halo acetyl, **C.** a sulphone, and **D.** a nitrile warhead, depicted in red, and first-point modification depicted in orange. This figure was produced by the authors.

Compound **93** (**QUB-00006-Int-07**) represents a significant advancement in the design of SARS-CoV-2 M^pro^ inhibitors (Figure 24A)^115^. This compound emerged from an optimisation process that started with a non-covalent inhibitor identified through in-silico studies, followed by meticulous refinement using molecular dynamics (MD) simulations and absolute binding free energy calculations. The resulting α, α-difluoro-keto inhibitor exhibits potent inhibitory activity against M^pro^, with an IC_50_ value of 0.83 µM. The formation of the covalent bond was confirmed using direct infusion electrospray ionisation mass spectrometry (ESI-MS). This technique provided direct evidence of the covalent adduct by detecting the mass shift corresponding to the addition of the inhibitor to the protease.

##### α-haloacetyl

A study aiming to find novel covalent inhibitors of SARS-CoV-2 M^pro^ via non-covalent and covalent docking of top ranked hits reported compound **94** as the most active inhibitor, with an IC_50_ of 8.50 µM (Figure 24B). The crystal structure (PDB: 7VVT)^116^ revealed the expected covalent bond, as well as other key non-covalent interactions^116^.

##### Sulphone

In a study employing both pharmacophore filtration and docking screening techniques, researchers identified compound **95** (**LY1**) as a potent covalent inhibitor of SARS-CoV-2 M^pro^ (Figure 24C)^117^. **95** exhibited a K_i_ value of 7.1 µM for M^pro^ inhibition. Its safety profile was investigated in Ver E6 cells using cell protection assay and confirming no cytotoxicity under the test conditions. An acute toxicity study and an 8-week toxicity study were also carried out, further confirming the safety profile of the inhibitor and concluding that the maximum tolerated dose is ∼ 1000 mg/kg, and the minimum lethal dose is 1300 mg/kg. Additionally, **95** demonstrated inhibitory activity against SARS-CoV-2 PL^pro^, with a K_i_ value of 16 µM, indicating its potential as a dual inhibitor. The covalent binding of **95** to M^pro^ was confirmed by crystallographic analysis (PDB: 7V1T)^118^.

##### Nitrile and aldehyde

Figure 24(D), depicts Compound **96** (**‘5103**), as one of the most potent SARS-CoV-2 M^pro^ inhibitors reported inhibitors in a docking-based study^119^. Bearing a nitrile warhead, it reported an IC_50_ of 20 µM. Compound **97** (**‘3620**) with an aldehydic warhead reported a much higher IC_50_ of 55 µM as shown in Figure 24(D). Compound **97** was optimised yielding compound **98** (**‘7021**) with a much better IC_50_ of 1 µM (Figure 24D). The covalent bond formation for compound **97** was confirmed via crystallography (PDB: 8DIB)^119^.

Looking at the examples discussed in this section, we can conclude that computer-aided drug design has limitations when it comes to identifying non-peptidomimetic covalent inhibitors for SARS-CoV-2 M^pro^, as the majority of the reported compounds exhibit high IC_50_ values and generally low potency. This suggests that alternative strategies may be more appropriate to discover more effective non-peptidomimetic inhibitors.

#### Non-peptidomimetic inhibitors from natural sources

Covalent inhibitors derived from natural sources offer a promising avenue for discovering novel therapeutics against SARS-CoV-2 M^pro^. These inhibitors are often found in plants, marine organisms, fungi, and other natural products, and are often, non-peptidomimetic in nature. Below are some examples of SARS-CoV-2 M^pro^ covalent inhibitors from natural sources found via *in-silico* and *in-vitro* screening campaigns.

**Figure 25.**
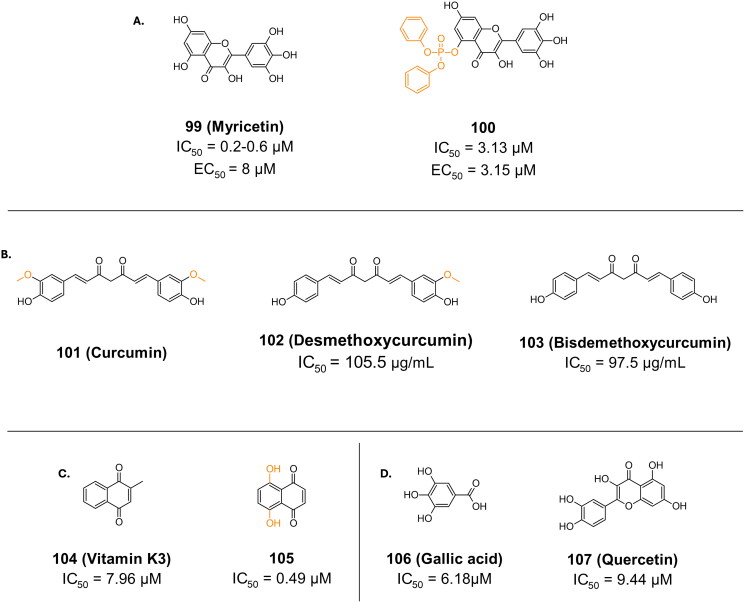
Chemical structures of non-peptidomimetics from natural sources as covalent inhibitors of SARS-CoV-2 M^pro^, with first-point modification depicted in orange. This figure was produced by the authors.

Compound **99** (**myricetin**), is a natural flavonoid with a Michael acceptor in its structure (Figure 25A), and an IC_50_ ranging from 0.2–0.6 µM, and an EC_50_ of 8 µM in Vero E6 cells^120^. Its CC_50_ was >200 µM. Its crystal structure (7DPP) reveals a covalent bond with Cys145 via the C6 in the pyrogallol moiety. This has been explained in literature by proposing that the pyrogallol undergoes oxidation to o-quinone, to allow for the attack of the thiolate from Cys145. Compound **100** is an analogue of **99** (**myricetin**), featuring a diphenyl phosphate at the 7-OH of **99** (Figure 25A)^120^. This had a noticeable effect on the EC_50_, lowering it to 3.15 µM in Vero E6 cells.

Compound **101** (**curcumin**) is another potent natural compound found via an *in-silico* based study, as a promising inhibitor of SARS-CoV-2 M^pro^ (Figure 25B)^121^. Its analogues compounds **102** (desmethoxycurcumin) and **103** (bisdemethoxycurcumin) were investigated for their potential inhibitory capacity, yielding IC_50_ values of 105.5 µg/mL and 97.5 µg/mL, respectively^122^.

Compound **104** (**vitamin K3**) is a quinonoid based inhibitor, found in an *in-vitro* screening campaign, reporting an IC_50_ of 7.96 µM (Figure 25C)^123^. Through optimisation, compound **105** had a 10-fold improvement in the IC_50_, reporting a value of 0.49 µM (Figure 25C)^124^. The covalent bond is thought to form with the quinone core, resulting in an irreversible bond via conjugate addition. This was proposed based on *in-silico* and kinetic studies.

In a study screening 60 clinically used antiviral herbal medicines for their potential to inhibit SARS-CoV-2 M^pro^, Lonicera japonica Flos (LJF) stood out for its robust anti-M^pro^ activity, with an IC_50_ value of 37.82 μg/mL^125^. MS analysis of LJF extract revealed the presence of at least 50 constituents, and through further chemo proteomic profiling, 22 constituents were identified as covalent modifiers of M^pro^. Among these, compound **106** (**gallic acid**) and compound **107** (**quercetin**) stood out as potent inhibitors of SARS-CoV-2 M^pro^, with IC_50_ values below 10 µM (Figure 25D). The inhibition mechanisms of gallic acid and quercetin were characterised using fluorescence resonance energy transfer, surface plasmon resonance, and covalent docking simulations. These analyses provided insights into the kinetics and mode of interaction of these compounds with SARS-CoV-2 M^pro^.

#### Non-peptidomimetic inhibitors from fragment-based drug design

Covalent fragments represent a promising strategy for the discovery and development of inhibitors against SARS-CoV-2 M^pro^. These small molecules, typically containing reactive groups capable of forming covalent bonds with specific residues within the protease’s active site, can serve as starting points for the development of more potent and selective inhibitors. Below are some examples of covalent fragments as SARS-CoV-2 M^pro^ inhibitors.

**Figure 26.**
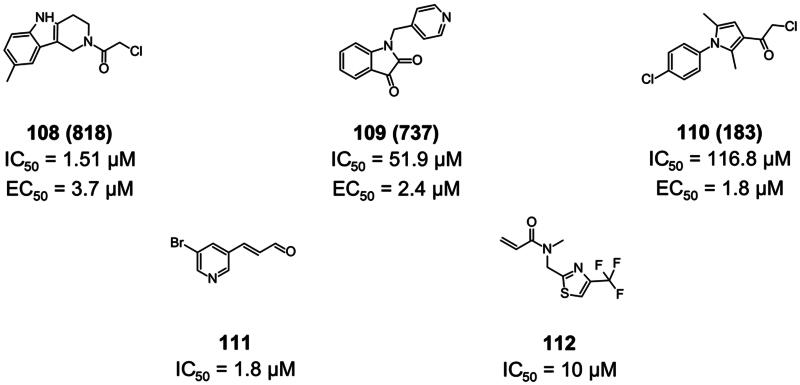
Chemical structures of non-peptidomimetics from fragment-based drug design as covalent inhibitors of SARS-CoV-2 M^pro^. This figure was produced by the authors.

In a recent study, compounds **108** (**818**), **109** (**737**), and **110** (**183**) emerged as significant fragment-like hits from an AI-based screening approach followed by *in-vitro* validation against SARS-CoV-2 M^pro^ (Figure 26)^126^. These compounds were tested for their inhibitory effects on the SARS-CoV-2 M^pro^ enzyme, as well as their antiviral activity in Vero E6 and Calu-3 cells. The IC_50_ values for these compounds were reported as 1.51 µM, 51.9 µM, and 116.8 µM, respectively. Compound **108**, despite containing a chloromethyl carbonyl moiety, was found to act through a reversible mechanism, indicating it functions as a non-covalent inhibitor. This surprising finding was confirmed through a reversibility assay. The reduced reactivity of this compound’s electrophilic warhead can be attributed to the presence of an amide bond to the gamma-carboline scaffold. Conversely, compound **109** (**737**) operates as a covalent inhibitor of M^pro^. The proposed mechanism involves the addition of the Cys145 thiol group to the ketone carbonyl at the C3 position of the compound. In terms of antiviral activity, compound **108** was effective in inhibiting SARS-CoV-2 replication in both Vero E6 and Calu-3 cells, with an EC_50_ value of 1.1 μM, and 3.7 µM. This close correlation underscores the compound’s potency as an inhibitor. Compounds **109** and **110**, while not effective in Vero E6 cells, showed significant activity in Calu-3 cells, with EC_50_ values of 2.4 μM and 1.8 μM, respectively. The effectiveness of these compounds in Calu-3 cells is particularly promising, given that this cell line serves as a human lung epithelial model, relevant for respiratory drug discovery.

Compound **111** is a pyridine-aldehyde fragment that was reported in a study investigating several reactive fragments as SARS-CoV-2 M^pro^ inhibitors^127^. At the end of the study, compound **111** with a mere molecular weight of 211 g/mol, had an IC_50_ of 1.8 µM. Compound **112** had the best IC_50_ of all investigated fragments in another study, reporting an IC_50_ value of 10 µM^128^. Its crystal structure confirms the covalent bond formation (PDB: 7WYM)^128^.

Due to their small molecular size and reactive characteristics, these fragments may exhibit promiscuity. For compounds **108**, **109**, and **110**, the study utilising AI-driven approaches included fine-tuning the model to generate compounds with potential activity against SARS-CoV-2 M^pro^. This was achieved by updating the model’s weights using a dataset of compounds with known biological activity against SARS-CoV-1 M^pro^. While the approach limited the novelty of the results, it also reduces the likelihood of identifying promiscuous compounds. In the case of compound **112**, the research group employed a method termed “quantitative irreversible tethering” (qIT), a high-throughput technique designed to identify selective covalent fragments that bind to a specific cysteine residue on a target protein. This approach enables effective hit prioritisation and reduces false positives and negatives by normalising the rate of protein modification based on the compound’s intrinsic reactivity. Although no *in-vitro* assays were performed to explicitly rule out compound promiscuity, the design of the methodology appears to account for this concern.

## Conclusion

The targeting of SARS-CoV-2 M^pro^ through both peptidomimetic and non-peptidomimetic covalent inhibitors represents a highly promising strategy in the fight against COVID-19. Peptidomimetic inhibitors, which closely mimic the natural substrates of M^pro^, have demonstrated significant potential due to their ability to form stable covalent bonds with the catalytic cysteine residue. Notable examples such as aldehydes, α-ketoamides, and ketones among others, have shown strong inhibitory activities and favourable binding profiles. Under this category, we have both reversible covalent bond forming inhibitors, as well as irreversible covalent bond forming inhibitors. Reversible covalent inhibitors, while generally safer than their irreversible counterparts due to reduced risk of idiosyncratic toxicities, may lack the potency required for targets like M^pro^, known for its challenging and expansive binding pocket. Irreversible inhibitors provide a robust solution by forming strong, permanent covalent bonds that are almost impossible to reverse, securing the compound within the active site, effectively overcoming challenges related to the pocket’s size. Importantly, these types of inhibitors necessitate rigorous safety evaluations despite the substantial structural differences between SARS-CoV-2 M^pro^ and human proteases, owing to the permanent and robust nature of the irreversible covalent bond.

On the other hand, non-peptidomimetic inhibitors offer diverse chemical scaffolds and mechanisms, including epoxides, repurposed drugs like AG7404, and even some promising examples from natural sources. These inhibitors not only exhibit potent inhibition of M^pro^ but also provide opportunities for targeting additional viral proteases, such as PL^pro^.

While peptidomimetic inhibitors excel in selectivity and potency by mimicking natural substrates but are hampered by poor bioavailability and metabolic stability. Conversely, non-peptidomimetic inhibitors might offer better stability and absorption profiles. However, despite their promising inhibitory activities, many of these inhibitors require further investigation beyond initial inhibition studies. There is a need to comprehensively evaluate their antiviral activity, selectivity profiles, and cytotoxicity. This deeper analysis is essential for advancing them as viable therapeutic candidates in combating SARS-CoV-2.

Overall, the progress made in both peptidomimetic, and non-peptidomimetic inhibitor development is encouraging. Future research should focus on optimising these compounds for improved efficacy, reduced toxicity, and enhanced pharmacokinetic properties. Moreover, the exploration of combination therapies could provide synergistic effects and help mitigate resistance development. Notably, the FDA-approved Pfizer drug, Paxlovid (nirmatrelvir/ritonavir), has set a precedent in this area. Paxlovid includes nirmatrelvir, a protease inhibitor that targets M^pro^, showcasing the real-world efficacy and potential of M^pro^ inhibitors in treating COVID-19. The success of Paxlovid underscores the importance of continued efforts in this domain and provides a valuable framework for the development of future antiviral therapies.

In conclusion, while covalent inhibitors targeting SARS-CoV-2 M^pro^ have shown promising *in-vitro* activity, a discrepancy often exists between their inhibitory (IC_50_) and antiviral efficacy (EC_50_) observed in cell-based assays. Factors such as solubility and cell permeability play crucial roles in determining the actual therapeutic potential of these compounds. The continued investigation and refinement of covalent inhibitors targeting SARS-CoV-2 M^pro^ will be crucial in developing effective antiviral treatments. These efforts not only contribute to managing the current pandemic but also lay the groundwork for rapid responses to future viral outbreaks from the same family. By integrating insights from structural biology, medicinal chemistry, and pharmacology, the scientific community can advance towards the goal of robust and broad-spectrum antiviral therapies.

## Data Availability

Data sharing is not applicable to this article as no new data were created or analysed in this study.
